# Direct Electrochemical CO_2_ Capture Using Substituted Anthraquinones in Homogeneous Solutions: A Joint Experimental and Theoretical Study

**DOI:** 10.1021/acs.jpcc.2c03129

**Published:** 2022-08-15

**Authors:** Corina Schimanofsky, Dominik Wielend, Stefanie Kröll, Sabine Lerch, Daniel Werner, Josef M. Gallmetzer, Felix Mayr, Helmut Neugebauer, Mihai Irimia-Vladu, Engelbert Portenkirchner, Thomas S. Hofer, Niyazi Serdar Sariciftci

**Affiliations:** † Linz Institute for Organic Solar Cells (LIOS), Institute of Physical Chemistry, Johannes Kepler University Linz, Altenberger Straße 69, 4040 Linz, Austria; ‡ Theoretical Chemistry Division, Institute for General, Inorganic and Theoretical Chemistry, University of Innsbruck, Innrain 80-82, 6020 Innsbruck, Austria; § Institute of Physical Chemistry, University of Innsbruck, Innrain 52c, 6020 Innsbruck, Austria; ∥ Institute of Applied Physics, Johannes Kepler University Linz, Altenberger Straße 69, 4040 Linz, Austria

## Abstract

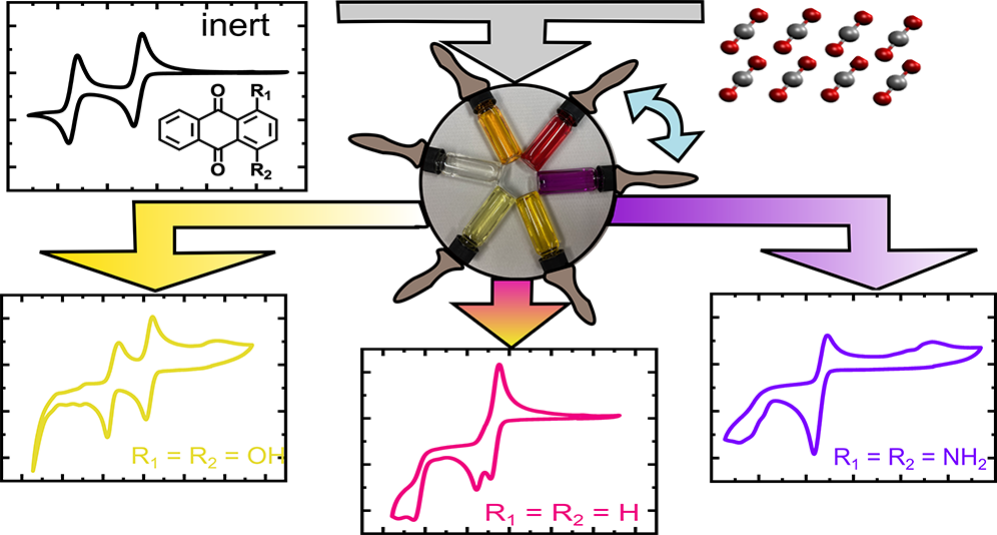

Electrochemical capture of carbon dioxide (CO_2_) using organic quinones is a promising and intensively studied alternative to the industrially established scrubbing processes. While recent studies focused only on the influence of substituents having a simple mesomeric or nucleophilicity effect, we have systematically selected six anthraquinone (AQ) derivatives (X-AQ) with amino and hydroxy substituents in order to thoroughly study the influence thereof on the properties of electrochemical CO_2_ capture. Experimental data from cyclic voltammetry (CV) and UV–Vis spectroelectrochemistry of solutions in acetonitrile were analyzed and compared with innovative density functional tight binding computational results. Our experimental and theoretical results provide a coherent explanation of the influence of CO_2_ on the CV data in terms of weak and strong binding nomenclature of the dianions. In addition to this terminology, we have identified the dihydroxy substituted AQ as a new class of molecules forming rather unstable [X-AQ-(CO_2_)_*n*_]^2–^ adducts. In contrast to the commonly used dianion consideration, the results presented herein reveal opposite trends in stability for the X-AQ-CO_2_^•–^ radical species for the first time. To the best of our knowledge, this study presents theoretically calculated UV–Vis spectra for the various CO_2_-AQ reduction products for the first time, enabling a detailed decomposition of the spectroelectrochemical data. Thus, this work provides an extension of the existing classification with proof of the existence of X-AQ-CO_2_ species, which will be the basis of future studies focusing on improved materials for electrochemical CO_2_ capture.

## 1 Introduction

In order to face the rising concentration of anthropogenic carbon dioxide (CO_2_) in the atmosphere, new and feasible strategies for carbon capture and utilization are required.^1−8^ Today the initial step of CO_2_ capture is mainly realized via a thermal sorption/desorption process,^3−5,9,10^ and electrochemical CO_2_ capture and release using organic carbonyl compounds is a new and promising alternative to these processes. Among those, quinones turned out to be the most promising material class due to their reversible reduction features involving two electrons. Although the history of such electrochemical CO_2_ capture dates back nearly four decades and is already well-reviewed,^11−14^ many highlighting publications just appeared in the past few years.

Already in 1984, Harada et al.^15^ reported the interaction of electrochemically reduced α,β-ketones with CO_2_ followed shortly afterwards by a report of Mizen and Wrighton^16^ focused on carbonate-like structures formed by the reaction of phenanthrenequinone with CO_2_. More detailed studies involving the influence of CO_2_ on cyclic voltammetry (CV) of different quinones were published by Simpson and Durand in 1990,^17^ by Nagaoka et al.^18^ in 1992, and by DuBois et al. in 1993.^19^ One major conclusion at that time was that the reduction potentials of quinones correlate qualitatively with its CO_2_ binding properties. In 2003, Scovazzo et al. reported a device capable of electrochemically concentrating CO_2_ from dilute 0.5% vol. to near purity using a benzoquinone derivative in ionic liquids (ILs).^20^ Concerning organic electrode materials for CO_2_ capture, our group identified and investigated the industrial pigment quinacridone,^21^ a naphthalene bisimide derivative,^22^ and an evaporated anthraquinone (AQ) thin-film^23^ as promising candidates for heterogeneous application. A very recent publication of Wang et al. describes an AQ-carbon nanotube composite as a heterogeneous electrode for electrochemical CO_2_ capture.^24^

In order to gain in-depth mechanistic insights, the majority of related publications focused on homogeneously dissolved quinone derivatives. Yin et al. investigated different quinones as mediators for Li-CO_2_ battery application involving exactly this electrochemical AQ-CO_2_ capture process.^25^ Gurkan et al.^26^ reported a design for an electrochemical cell for CO_2_ separation based on dissolved naphthoquinone (NQ) in ILs, and in 2021, Tam et al.^27^ provided a detailed study on methyl substituted NQs for CO_2_ capture, including UV–Vis spectroelectrochemistry (SEC). Recently, Hatton and co-workers published an in-depth study on the nature of the CO_2_-quinone binding in relation to the CO_2_ binding strength mentioned above by comparing various different quinones, but without any hydrogen-bonding substituents.^28^ The same group also developed several prototype cells for direct air capture of CO_2_ based on AQ polymers.^29−31^ It has been reported that oxygen (O_2_) binding and electrochemical O_2_ reduction by these AQ-polymers are crucial side-reactions to be considered.^29,32^ Very recently, Bui et al. investigated the impact of multiple substitutions involving several AQ derivatives with CV and related their findings regarding electrochemical potentials with results obtained via density functional theory (DFT) calculations.^33^

Besides the use of quinones for electrochemical CO_2_ capture, also numerous applications of quinones for metal-ion batteries^34−40^ as well as redox-flow batteries^41−43^ are reported. As quinones in electrochemical applications are often limited due to reductive dissolution from the electrode surface,^44^ metal-ion battery research is focused on the prevention of this dissolving process via polymerization or tuning of the substrate-quinone binding strength.^40,45^

One major aspect regarding the stabilization of reduced quinone species on the electrode surface is the influence of inter- and intramolecular hydrogen bonds. Already in 1984, Ashnagar et al.^46^ described the systematic influence of the position of hydroxy groups in AQs on their electrochemical properties, which was refined via computational studies by Shamsipur et al.^47^ and Schwan et al.^42^

It has been shown that in addition to intramolecular hydrogen bonding, the presence of water^48,49^ as well as other protic solvents and additives^50,51^ have been reported to have a strong influence on the electrochemical properties of substituted quinones. Barlow and Yang recently even suggested a promising, controlled tuning of the electrochemical features and CO_2_ capture abilities of quinones via proton-donating additives.^51^

**Figure 1:**
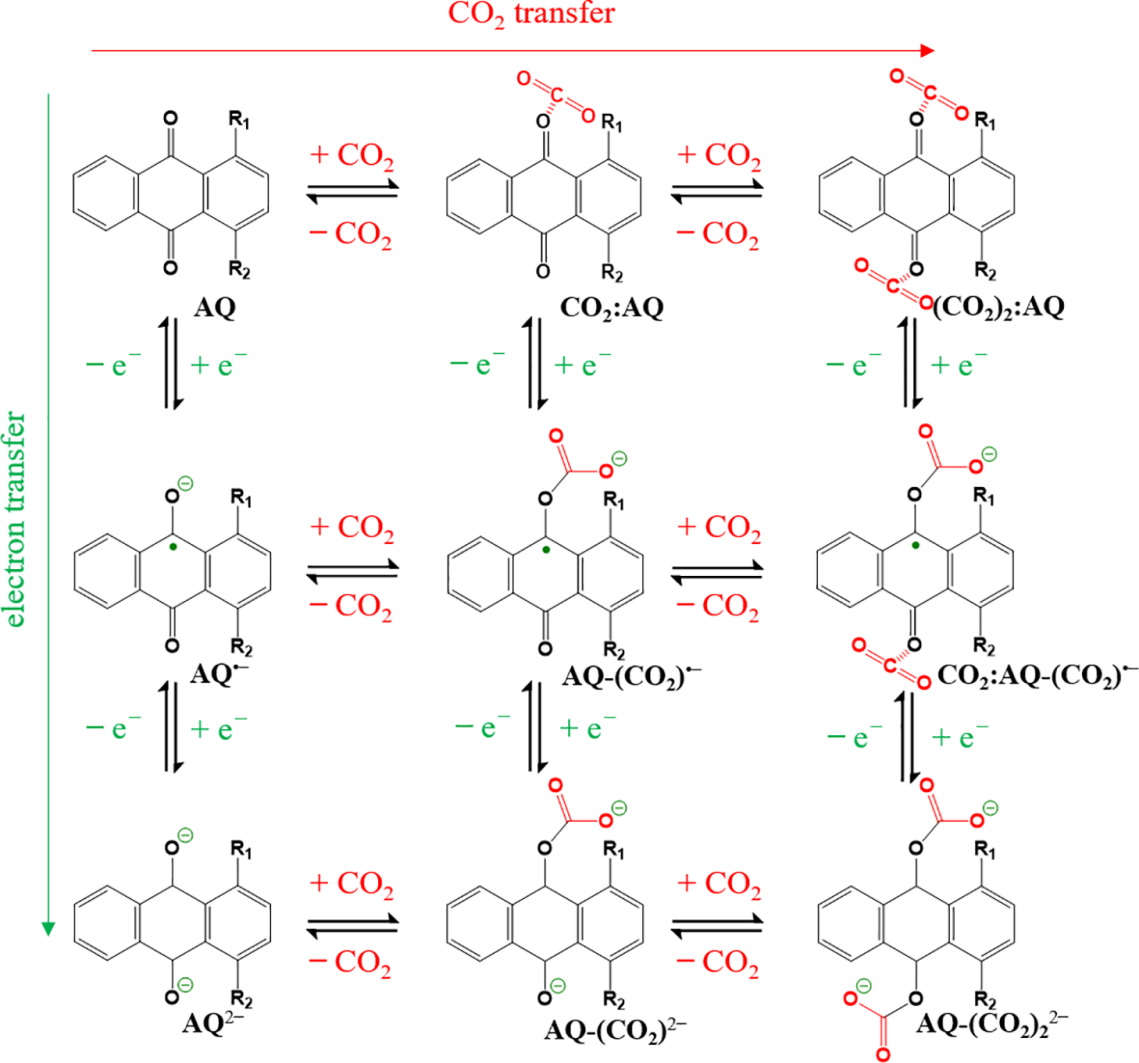
Proposed mechanism for the products of electrochemically reduced AQs in the presence of CO_2_. The vertical transitions represent electron transfer reactions, while horizontal transitions refer to CO_2_ transfer reactions. The substituents R_1_ and R_2_ reflect the different AQ derivatives considered throughout this work being either: −H, −NH_2_, or −OH. Scheme created upon consideration of the proposed reaction scheme from refs (26−28).

In a recent joint experimental and theoretical publication, the influence of two substituent groups involving hydrogen bonds—amino and hydroxy groups—on the electrochemical behavior under inert conditions has been studied.^52^ Based on these results, a set of mono- and disubstituted derivatives of AQs carrying amino or hydroxy groups in the β-position relative to the carbonyl groups have been selected, which are represented via the side groups R_1_ and R_2_ in Figure 1. These AQ derivatives were investigated in terms of their electrochemical CO_2_ capture properties in homogeneous solutions via CV and UV–Vis SEC as well as theoretically via density functional tight binding (DFTB) calculations. The latter supports the experimental investigation by providing a structural model to understand the interaction between the respective AQ derivatives and CO_2_ during the reduction process. Based on our preliminary results and literature reports, we propose that CO_2_ is either covalently bound or forms a nonbonded coordination toward the AQ-derivatives, as depicted in Figure 1.

To investigate the most probable binding motif according to the proposed literature scheme shown in Figure 1, the respective calculated UV–Vis spectra are compared with their experimental counterparts. By augmenting the experimental findings via computational investigations, our results reveal unprecedentedly that amino and hydroxy substituents have an opposite effect on the single-reduced radical and dianionic species with CO_2_, which is essential for future studies targeting tunable quinone-CO_2_ binding properties.

## 2 Experimental Section

### 2.1 Materials Purification

Throughout this work, all six possible derivatives of mono- and disubstituted AQ’s with hydrogen, amino, or hydroxy groups (as R_1_ and R_2_ in Figure 1) in the 1 and 4 positions were investigated. Each of the six AQs was purified from the as-purchased samples (Sigma-Aldrich) using a quartz tube in vacuum at a pressure below 1 × 10^–5^ mbar. Two borosilicate glass tubes were fused by flame and inserted into the quartz tube; they served as a confinement of the source material and as a means of recovering the sublimed material at the end of the sublimation process. Each material investigated in this work was sublimed within a 24 h period at a particular temperature ranging from 150 to 200 °C. Moreover each of the six AQ derivatives (X-AQ) was purified by two such successive temperature gradient sublimations in order to increase the purity, as thoroughly described in recent publications.^53,54^

### 2.2 Optical Characterization

UV–Vis spectra of each material were recorded in a range of 300 to 700 nm on a Cary 3G UV–Visible spectrophotometer. Depending on the absorption coefficient of the materials, a 100 or 50 μM AQ derivative solution in acetonitrile (MeCN, Roth) was used. The wavelength of the absorption maximum of the respective derivate was used as the excitation wavelength for the following photoluminescence (PL) measurements.

For the PL measurements the diluted solutions (100 and 10 μM) were filled into a quartz glass PL cuvette (Hellma) and analyzed with a PTI QuantaMaster 40 spectrofluorometer using a dual monochromator setup on the excitation as well as the emission channel. In the excitation channel, the slit widths were set to 2 mm, while in the emission channel, slit widths of 1 mm were used. To block light from lower orders of diffraction, an additional 320 nm long-pass filter was placed in the excitation light path.

### 2.3 Electrochemical Characterization

The electrochemical measurements were performed in a one compartment cell, consisting of a glass vial with a platinum wire as counter electrode (CE), an Ag/AgCl quasi reference electrode (QRE), and a glassy carbon (GC, PalmSens) working electrode (WE). Unless stated otherwise, all potentials reported in this work refer to the standard hydrogen electrode (SHE) and were calculated from calibration of the QRE with ferrocene (Sigma-Aldrich) using the literature value of +620 mV vs SHE in MeCN.^55^ To obtain a 2 mM anthraquinone derivative (X-AQ) solution, an according amount of the respective material was dissolved in a 0.1 M solution containing tetrabutylammonium hexafluorophosphate (TBAPF_6_, Sigma-Aldrich) as electrolyte in MeCN (Roth).

For the electrochemical characterization using CV, a Vertex One Ivium potentiostat/galvanostat was used. All CV curves investigating the oxidation and the reduction of the materials were recorded at a scan rate of 200 mV s^–1^. The CV scans only investigating the cathodic regime under N_2_ and CO_2_ saturated conditions were measured at a scan rate of 100 mV s^–1^. To reach a CO_2_ saturated environment, the solution was purged with CO_2_ (Linde Gas) for 15 min, prior to the measurements.

In order to compare the experimentally determined redox features among each other and with literature, the half-step potentials *E*_p/2_ were determined. Thereby, the *E*_p/2_ corresponds to the potential of a redox feature, where half of the peak current was established.

For determination of the number of electrons transferred in CV studies, the current–time curves were integrated and the charges (in μC) compared. For better clarity, these electrical charges were assigned to shaded areas in the current–potential graphs.

### 2.4 Spectroelectrochemistry

For the spectroelectrochemical measurements, a special thin-layer-quartz glass cuvette (Basi) equipped with a platinum mesh as WE and filled with a 1 mM X-AQ solution in 0.1 M TBAPF_6_/MeCN was used. The electrochemical reduction was performed with the Vertex One Ivium potentiostat/galvanostat. Here, the potential was decreased stepwise by 200 mV steps every 37 s. During the hold potential phases, UV–Vis spectra were recorded with a Cary 3G UV–visible spectrophotometer from 300 to 700 nm at a scan rate of 1515 nm/min. In all the main figures, the absorbance values of the recorded spectra are illustrated.

### 2.5 Computational Details

#### 2.5.1 Calculation Settings

Considering the advantageous cost-accuracy ratio and the good performance in previous studies focused on AQ-graphite interactions^40^ and the electrochemical potential of different AQ derivatives,^52^ self-consistent charge density functional tight binding (SCC DFTB)^56,57^ in conjunction with the 3ob parameter set^58^ and D4 dispersion correction^59−61^ was applied. To take the influence of solvation effects into account, the calculations were performed considering acetonitrile with two different kinds of implicit solvation models: the generalized Born solvent area model^62^ (GBSA) (relative permittivity ε = 37.5, molar mass *M* = 41.05 g mol^–1^, and density ρ = 0.786 kg L^–1^, gfn2 parameter set) reported by Grimme et al.^63,64^ and the conductor-like screening model (COSMO)^65,66^ (relative permittivity ε = 37.5, molar mass *M* = 41.05 g mol^–1^, and density ρ = 0.786 kg L^–1^). However, the influence of the electrolyte salts could not be considered in this approach.

All calculations were executed using the program DFTB+ (v. 21.1).^67^ To reproduce the experimental setting and the various kinds of interactions, three different systems were considered for each X-AQ derivative, namely (i) covalently bound CO_2_, (ii) coordinated CO_2_, and (iii) the respective X-AQ derivative in the absence of CO_2_, according to the proposed structures in Figure 1. The calculations were carried out for different charged states being 0, −1, and −2 to the individual steps in the reduction reactions. All experimentally measured derivatives, AQ, 1,4-NH_2_-AQ, 1-NH_2_-AQ, 1-NH_2_-4-OH-AQ, 1-OH-AQ, and 1,4-OH-AQ, have been considered in the calculations, thereby taking all possible *syn*- and *anti*-conformations associated with the hydroxy substituents into account, yielding a total of 11 target systems. (Compare to Figure 1 for the according substitution pattern with respect to the groups R_1_ and R_2_.) Visualization of the resulting structures was carried out using VMD.^68^

#### 2.5.2 Geometry Optimization

**Figure 2:**
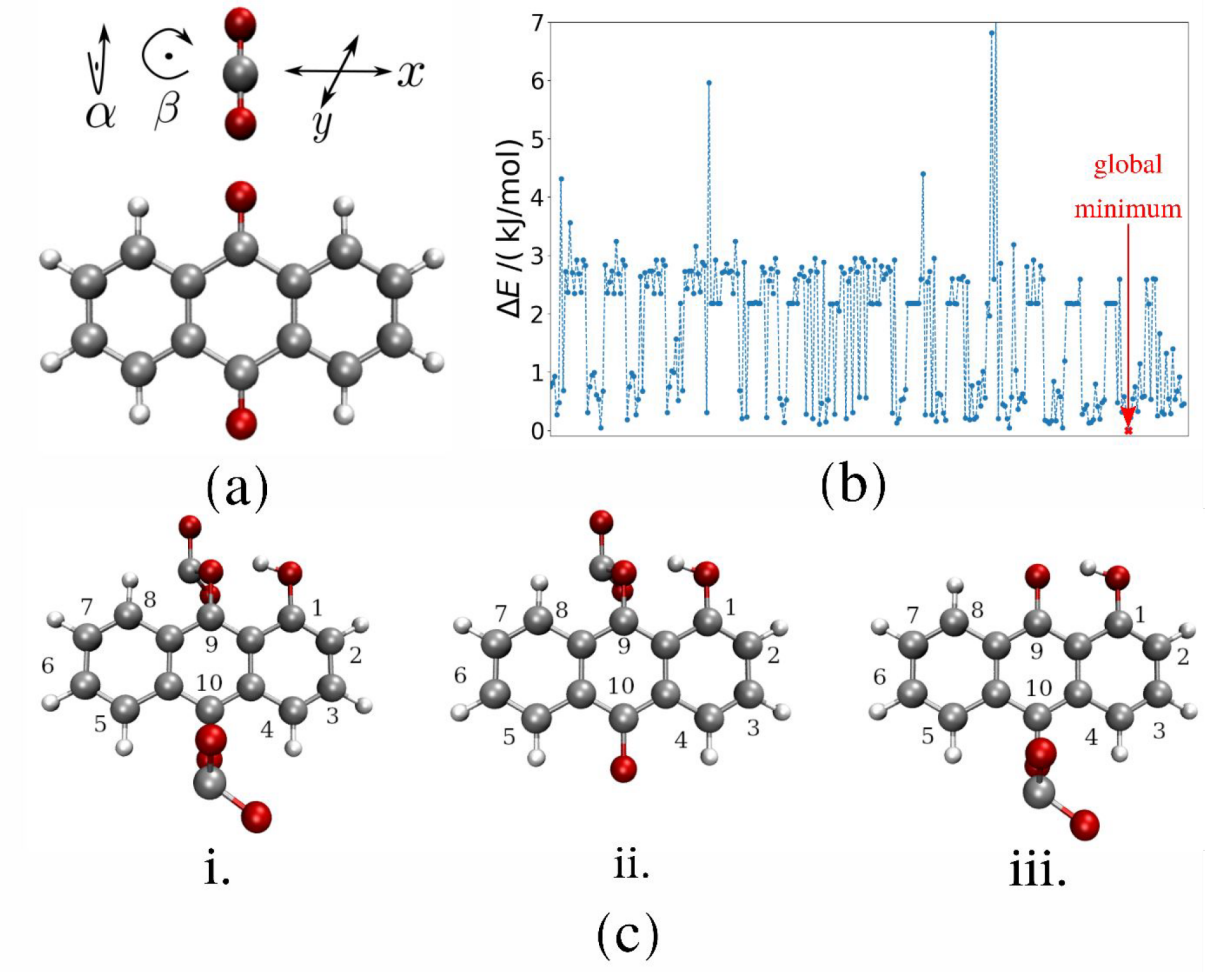
(a) Illustration of the starting geometries prepared to probe the ideal binding motif for the nonbonded CO_2_-AQ systems via basin hopping optimization. The CO_2_ is rotated from the initial position in the range from 0.0° to 90.0° in α- and β-directions and shifted from 0.0 to 5.0 Å and from 0.0 to 2.0 Å in *x*- and *y*-directions, respectively. (b) Energy profile obtained from a total of 288 individual energy minimizations carried out at different starting configurations enabling the location of the global minimum at the example of the neutral AQ-CO_2_ system in implicit solvent (GBSA model). (c) Illustrating of the starting geometries in case of CO_2_ covalently bound to 1-OH-AQ (*syn*): (i) dual bound CO_2_ molecules, (ii) single CO_2_ molecule bound at the 9-C=O, and (iii) at the 10-C=O positions. Color code of the atoms: H, white; C, gray; and O, red.

Depending on the interaction motif, different approaches to identify the minimum configuration were applied:

(i) Coordinative interaction of CO_2_: To identify the ideal interaction motif between CO_2_ and the AQ-derivatives, basin hopping global optimization^69^ was carried out, thereby executing a series of individual geometry optimizations at SCC DFTB/3ob/DFTD4 level. The starting geometries were constructed by rotating the CO_2_ from 0.0° to 90.0° along the α- and β-directions as well as by shifting it from 0.0 to 5.0 Å and from 0.0 to 2.0 Å in *x*- and in *y*-directions, respectively, (see sketch in Figure 2a) yielding a total of 288 possible initial geometries. The respective configuration was selected as a valid starting structure for the energy minimization, if a minimum distance of 1.0 Å was maintained between every atom of the respective X-AQ derivative and the CO_2_ molecule. The result of this optimization study yields a set of local minimal structures that represents a discretized potential energy landscape of the coordinated X-AQ-CO_2_ system. This approach enables the identification of several basins of attraction and the respective global minimum from the associated energy profile^69^ (see Figure 2b for the exemplary case of AQ-CO_2_).
  The outlined procedure was applied for all AQ derivatives and charged states in conjunction with the GBSA solvation model, except the −2 charged state of the 1,4-diaminoanthraquinone, 1-aminoanthraquinone, 1-amino-4-hydroxyanthraquinone and 1,4-dihydroxyanthraquinone (*syn/syn* or *s/s*). In the latter cases, convergence problems of the SCC DFTB calculations were encountered, and a preoptimization employing the GFN2-xTB^70−73^ approach in implicit acetonitrile (GBSA model)^63^ had to be carried out. Next, the optimized geometries were reoptimized at the SCC DFTB/3ob/DFTD4 level. Due to the good comparability of the two kind of solvation models (GBSA and COSMO), the basin hopping optimization was only applied in the GBSA case. The resulting global minimum structures obtained using the GBSA solvation model were then considered as a suitable initial structure for the calculation employing the COSMO approach, starting with a reoptimization followed by the calculation of the respective UV–Vis spectra. Since the latter proved to be superior over the GBSA model, only results obtained via the COSMO approach are shown.
(ii) Covalently bound CO_2_: In the case of the covalently bound CO_2_ systems, three different bonding situations were taken into account. The formation of a covalent O–C bond at either one or both carbonyl groups in 9- and 10-position of the respective AQ derivative is considered, unless symmetry of the substitution leads to two identical CO_2_ adducts (see Figure 2c). Since, the covalent situation is expected to correspond to the fully reduced species, only the −2 charged state was taken into account.^18,19,27,28^ Test calculations of the covalently bound X-AQ-CO_2_ system at neutral conditions and the −1 charged species indeed resulted in a dissociation of carbon dioxide, confirming that a nonbonded interaction motif is preferred in the oxidized and the −1 charge state as expected. All systems were optimized by SCC DFTB/3ob/DFTD4 in conjunction with the GBSA and COSMO solvation models.
(iii) Absence of CO_2_: In addition to studying the different X-AQ-CO_2_ interaction motifs, the systems were computed in absence of CO_2_ to compare the measured and calculated UV–Vis spectra under N_2_ saturated conditions. In this case, geometry optimizations at SCC DFTB/3ob/DFTD4 level were employed for all systems, solvation models, and charged states.

#### 2.5.3 Excited-State Calculations

Following the determination of the optimized X-AQ-CO_2_ geometries, the calculation of the UV–Vis spectra was carried out at the SCC DFTB/3ob/DftD4 level using the electronic dynamics framework as implemented in the DFTB+ package within the Ehrenfest ansatz.^74^ In this framework, a Dirac δ-type perturbation is applied to the ground-state density matrix, which is subsequently propagated in time. A total of 20,000 time steps with a length of 0.2 au (approximately 8.268 fs) were carried out employing a field strength of 0.001 V/Å in the perturbation. The Fourier transform of the resulting dipole moment evolution along the three principal directions enables the calculation of the associated UV–Vis spectral data. In order to achieve a line broadening that corresponds to that observed in the experimental investigations, a window function with a damping constant of 3.7 fs was applied in the Fourier transform step.

## 3 Results and Discussion

### 3.1 Electrochemical Results

**Figure 3:**
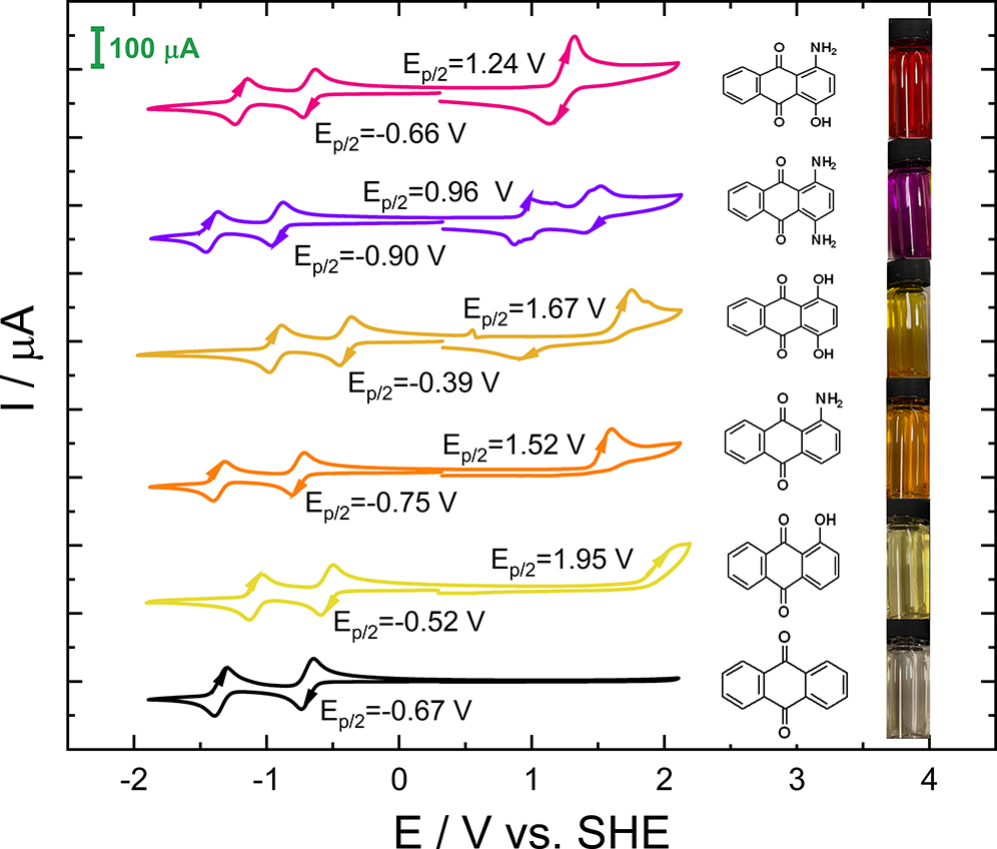
Chemical structures and CV curves of 2 mM solutions of the six X-AQ derivatives recorded in 0.1 M TBAPF_6_ in MeCN at a scan rate of 200 mV s^–1^. On the right side, the colors of solutions of the corresponding compounds in MeCN are depicted.

The redox behavior of all six AQ molecules considered within this study obtained from the CV measurements is comparatively shown in Figure 3.

The CV curve in Figure 3 of unsubstituted AQ shows two reversible reduction peaks, which correspond to the two well-known single electron reduction steps of the AQ derivatives, leading to a dianionic X-AQ^2–^ species with an increased charge density at the reduced carbonyl groups. In accordance with the optical band gap of 3.9 eV for the unsubstituted AQ (see Supporting Information Figure S1 for absorption and PL data), no electrochemical oxidation feature was observed in this measurement. As pointed out earlier, the objective of this work is to study and understand the influence of amino and hydroxy substitutions in relation to the unsubstituted AQ. Comparing the reduction peaks of the amino- and hydroxy-substituted AQs with the unsubstituted one reveals that amino groups lead to a cathodic shift, while hydroxy groups cause an anodic shift. Although both groups are considered to increase the electron density of a mesomeric system, this behavior can be explained by means of intramolecular hydrogen bonding as recently reported by Gallmetzer et al.^52^ In another previous work, it was demonstrated that the substitution of AQ with an alkoxy group results in a cathodic shift in the reduction potential.^44^ These shifts are more pronounced for the disubstituted molecules compared to their monosubstituted counterparts. Interestingly, in the case of 1-NH_2_-4-OH-AQ, these two effects level out so that its first-reduction peak is located at nearly unchanged potential compared to the unsubstituted AQ. However, the second-reduction peak displays a slight anodic shift in comparison to the pristine AQ. Moreover, for each AQ derivative considered in this study, at least one oxidation peak was observed, which is an indication that both types of substitution facilitate molecular oxidation. Thereby, only in the case of the disubstituted analogues, a corresponding re-reduction peak could be identified, which can be attributed to the favorable 1,4-position (*para*-position). In the case of 1,4-NH_2_-AQ, two clearly separated oxidation peaks were observed, where both show reversible re-reduction features which is described in further detail in Gallmetzer et al.^52^

**Figure 4:**
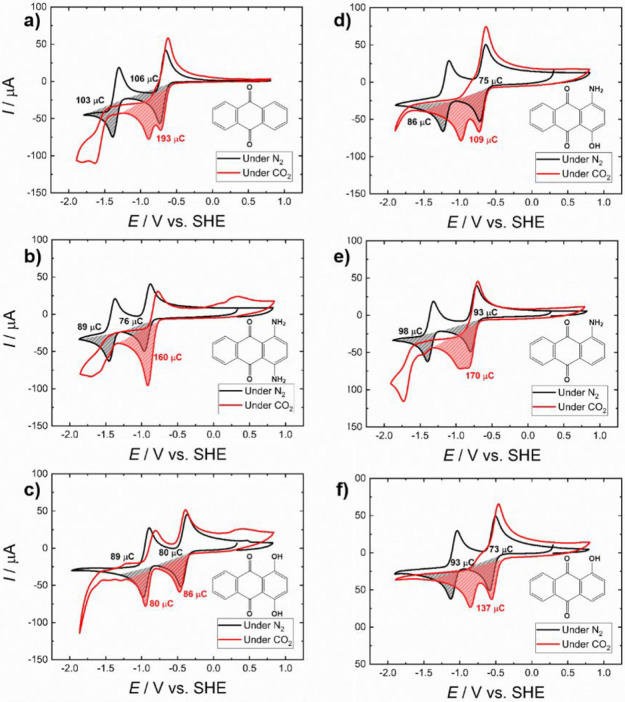
CV graphs of 2 mM solutions in 0.1 M TBAPF_6_ in MeCN at a scan rate of 100 mVs^–1^ under N_2_ conditions (black line) and under CO_2_ conditions (red line). (a) unsubstituted AQ, (b) 1,4-NH_2_-AQ, (c) 1,4-OH-AQ, (d) 1-NH_2_-4-OH-AQ, (e) 1-NH_2_-AQ, and (f) 1-OH-AQ. The CV curves under CO_2_ conditions refer to stable conditions after 20 CV cycles. The shaded areas under the curves comparing the reduction peaks illustrate the corresponding electric charges, which were determined via integration of the current–time graphs.

In a next step, the impact of CO_2_ saturated conditions on the CV measurements of the six substituted AQ derivatives was investigated, as depicted in Figure 3. According to several literature studies, an effect of CO_2_ on the redox properties has already been reported for various naphtho-^17,26,27,75^ and AQ^16,17,25^ compounds in aprotic solvents as well as for AQ in aqueous solution.^23^ In Figure 4, a comparison between CV cycles recorded under inert (N_2_) and CO_2_ saturated conditions is given.

The CV curves obtained for unsubstituted AQ under both inert (N_2_) and CO_2_ saturated conditions are shown in Figure 4a and are in good agreement with previous literature reporting two merged or partially merged reduction peaks with a corresponding reoxidation feature.^17,25−27^ Furthermore, in some experiments, an additional irreversible reduction peak at around −1.50 V was observed in the presence of CO_2_. Although the CV curves of the disubstituted AQ derivatives under inert conditions are comparable to unsubstituted AQ, under CO_2_ saturated conditions, a number of notable differences were observed. The dihydroxy anthraquinone (Figure 4c) shows a nearly unchanged behavior with slightly increased current upon CO_2_ addition, while in the case of the diamino anthraquinone (Figure 4b), the first-reduction peak shows roughly twice the current compared to inert conditions, and, similar to the unsubstituted AQ, an additional reduction peak at −1.47 V appears. The CV graphs for the monosubstituted analogues and 1-NH_2_-4-OH-AQ are shown in Figure 4d–f, and a summary of the reduction potentials of all six compounds is depicted in the Table 1.

**Table 1:** Summary of the Half-Step Reduction Potentials *E*_p/2_ of the X-AQ Derivatives under N_2_ Saturated Conditions and CO_2_ Saturated Conditions and Calculated CO_2_ Binding Constants *K*_b,CO_2__

	*E*_p/2_ (under N_2_)		*E*_p/2_ (under CO_2_)			
compound	1st	2nd	1st	2nd	3rd	log(*K*_b,CO_2__)
AQ	–0.67 V	–1.32 V	–0.66 V	–0.86 V	–1.50 V	9.0
1-OH-AQ	–0.52 V	–1.06 V	–0.49 V	–0.94 V	–	5.1
1,4-OH-AQ	–0.39 V	–0.91 V	–0.40 V	–0.90 V	–	1.1
1-NH_2_-AQ	–0.75 V	–1.34 V	–0.73 V	–	–1.64 V	8.3
1,4-NH_2_-AQ	–0.90 V	–1.39 V	–0.89 V	–	–1.47 V	9.7
1-NH_2_-4-OH-AQ	–0.66 V	–1.17 V	–0.67 V	–0.96 V	–	4.8

One major conclusion from the comparative potential results, shown in Table 1, is that the first-reduction potential is unaffected by the presence of CO_2_ for each of the six derivatives considered. Depending on the type and number of substituent groups, the second-reduction peak under CO_2_ saturated conditions is strongly affected. Qualitatively, the CV graphs in Figure 4 suggest either a shift of the second-reduction peak toward the first and in some cases an additional third-reduction feature or a cathodic shift of the second-reduction peak and rise of an additional peak between the first and the second one. Considering the qualitative number of charges in each CV peak, it can be stated that the second-reduction peak is shifted to a more positive potential, which is also in agreement with previous works.^17,18,28^

According to literature,^19,28,51^ the binding constant of the CO_2_:X-AQ^2–^ adduct, *K*_b,CO_2__, can be calculated from the shift of the second-reduction peak under N_2_ in comparison to CO_2_ saturated conditions Δ*E*_p*,*__2_ using the formula shown in eq 1:
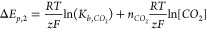
1where *R* is the universal gas constant, *T* the temperature, *F* the Faraday constant, [CO_2_] the concentration of dissolved CO_2_, and *z* the number of electrons transferred in the reduction of X-AQ^•–^ to X-AQ^2–^, which is 1. The number of CO_2_ molecules bound per X-AQ^2–^ molecule *n*_CO_2__ is, in accordance to previous literature,^19,51^ assumed to be 1 for the *K*_b,CO_2__ of the first CO_2_ molecule attached. In our CO_2_-saturated MeCN conditions at atmospheric pressure, a value of [CO_2_] of 0.28 mol_CO_2__ L_MeCN_^–1^ as given in literature was employed.^76^ The shift of the second-reduction peak Δ*E*_p*,*__2_ is calculated according to eq 2:
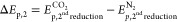
2

According to the log(*K*_b,CO_2__) values in Table 1, the 1,4-OH-AQ derivative demonstrating almost no discernible potential shift also displays the smallest value of 1.1, while in the 1,4-NH_2_-AQ case, the fully merged 2-electron feature under CO_2_ corresponds to the largest log(*K*_b,CO_2__)-value of 9.7. A more detailed analysis and discussion of this trend considering the binding constants *K* with CO_2_ and DFTB calculated results can be found in section 3.3.

When performing CV studies under CO_2_ saturated conditions, a significant increase in current occurs in the highly cathodic region during the first cycles that vanishes over the CV cycle number. Because all other peaks remain unchanged, the 20th cycle for the CO_2_ measurements has been included in Figure 4 in order to show the stable and reproducible scans. In the case of unsubstituted AQ, 1-NH_2_-AQ and 1,4-NH_2_-AQ, a third-reduction peak at potentials more negative than −1.5 V was observed, which is not present in blank GC electrode CV experiments (Figure S2). As these reduction features are irreversible, but no CO_2_ reduction product could be detected via gas chromatography, an oligomerization of AQ and CO_2_, beyond the structures depicted in Figure 1, might occur. Nevertheless, a detailed analysis of this CV feature observation is beyond the scope of this work and subject to future studies.

### 3.2 Spectroelectrochemistry

**Figure 5:**
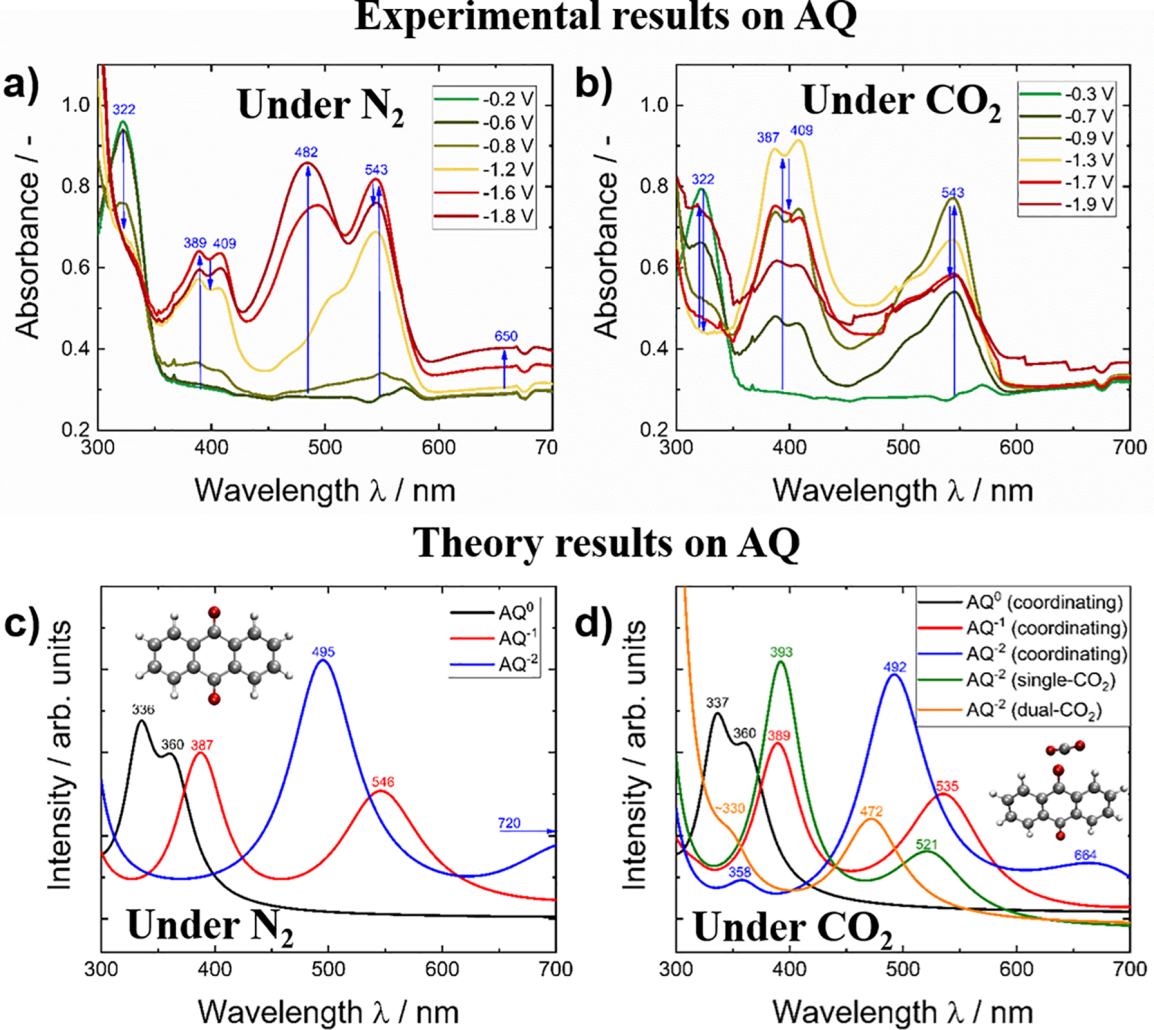
Experimental SEC graphs of unsubstituted AQ during reduction under (a) N_2_ conditions and (b) CO_2_ saturated conditions. The lower graphs show DFTB calculated spectra (c) under inert conditions and (d) in the presence of CO_2_. The insets refer to the geometry optimized structures of AQ (c) and to the coordinated CO_2_:AQ^•–^ structure (d).

To provide further insight into the physicochemical properties of electrochemical CO_2_ capture, the changes in the UV–Vis absorption spectra during reduction of unsubstituted AQ were recorded via SEC under inert (N_2_) and CO_2_ saturated conditions, respectively. A comparison to the theoretically calculated absorption spectra of the involved reduced AQ species, is illustrated in Figure 5.

The experimental spectra under N_2_ saturated conditions are in good agreement with our previous results of AQ recorded in inert aqueous conditions.^23,77^ Comparison of the absorbance plots of AQ under inert conditions depicted in Figure 5a,c shows a moderate deviation for the neutral species when comparing the experimental and theoretical spectra. These spectral changes follow the 2-electron reduction scheme depicted on the very left reaction pathway in Figure 1. A highly remarkable fact is that the calculated spectra for the single and double-reduced AQ species under inert conditions show excellent agreement with their experimental counterparts obtained via SEC. The single-reduced AQ^•–^ radical was measured to have twin bands at 389 and 409 nm and a single absorption signal at 543 nm, which is in good agreement to literature reports.^78,79^ The fully reduced AQ^2–^ species was determined to have a distinct absorption band at 482 nm and a broad absorption band at around 650 nm. The bands measured for both species show a very good correlation with the computed spectra shown in Figure 5c with only minor shifts in the wavelength as compared to the calculated band absorptions. Upon changing to a CO_2_ saturated system, at a first glance, no pronounced change was observed for the calculated and experimental spectra as the position of the absorption bands was only marginally shifted. At closer investigation, the bands at 482 and 650 nm of AQ^2–^ are missing under CO_2_ conditions. This can be understood in terms of the proposed reaction of the reduced AQ species with CO_2_ forming [AQ-(CO_2_)_2_]^2–^, as depicted in Figure 1. The calculated absorption bands for AQ^2–^ with a single bound CO_2_ are 393 and 521 nm, whereas the AQ^2–^ bound to two CO_2_ molecules is expected to show absorption features at 330 and 472 nm (see Figure 5d). Although the SEC under CO_2_ lacks the distinct band observed at 482 nm for N_2_ conditions, a shoulder in this wavelength range was observed which is in good agreement with the predicted absorption regime of AQ-CO_2_ adducts. Under both N_2_ and CO_2_ conditions, the absorption of AQ at 322 nm initially vanishes toward increasing reductive potentials. The reappearance of the absorption band at around 330 nm under CO_2_ at −1.9 V suggests the formation of the dual CO_2_ adduct. Interestingly, the calculated absorption for the double-reduced AQ with two bound CO_2_ molecules at 472 nm is very close to the feature reported for evaporated AQ thin-films upon electrochemical reduction under CO_2_ saturated conditions in aqueous solution at 440 nm.^23^ The full trend of the change in absorbance of each band in case of the unsubstituted AQ under N_2_ and CO_2_ as well as the Δ absorbance spectra are depicted in Figure S3.

In addition to the spectral changes upon reduction of unsubstituted AQ under inert and CO_2_ saturated conditions, the spectroelectrochemical graphs during reduction of 1,4-NH_2_-AQ in comparison with the calculated spectra are shown.

**Figure 6:**
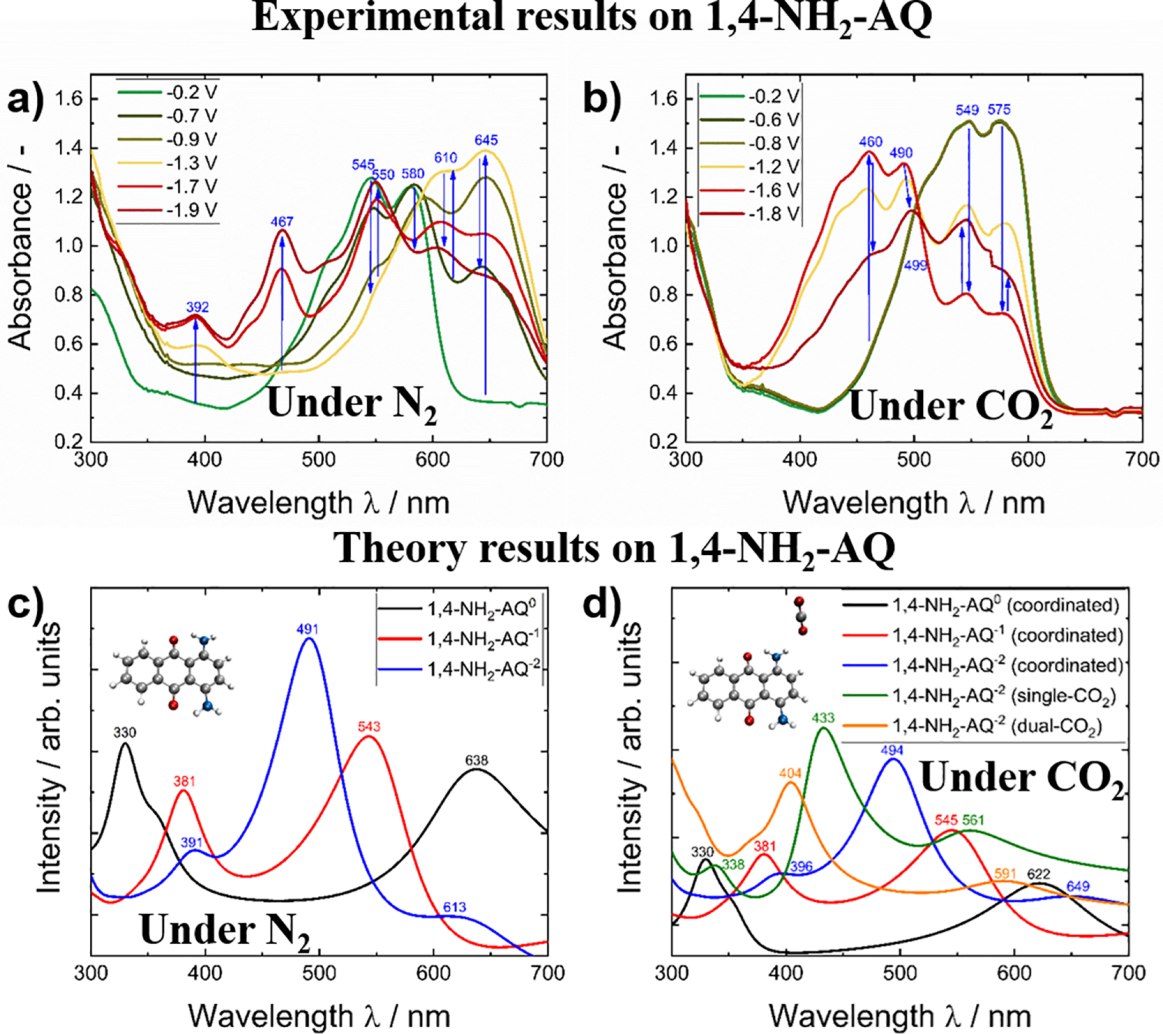
SEC graphs of 1,4-NH_2_-AQ during reduction under a) N_2_ conditions and under b) CO_2_ saturated conditions. The lower graphs show DFTB calculated spectra under c) inert conditions and d) in the presence of CO_2_. The insets refer to the geometry optimized structures of 1,4-NH_2_-AQ (c) and to the coordinated CO_2_:1,4-NH_2_-AQ^•–^ structure (d).

Comparing the experimental UV–Vis absorption features in Figure 6 of neutral 1,4-NH_2_-AQ with the calculated spectra reveal a larger deviation compared to the unsubstituted AQ case in Figure 5 as the predicted low-energy transitions were bathochromically shifted in the calculations compared to the experimental values.

The absorption bands at 392 and 467 nm arising upon electrochemical reduction under inert conditions (See Figure 6a) can be clearly assigned to the formation of the 1,4-NH_2_-AQ^•–^ radical and the 1,4-NH_2_-AQ^2–^ dianionic species. A precise assignment in the regime above 500 nm is not possible, as the decreasing absorption intensities associated to the neutral species overlap with the rising bands of the reduced species. The fading and reappearing of the band at 545–550 nm can be most likely attributed to the 1,4-NH_2_-AQ^•–^ radical. As shown in Figure 6b, under CO_2_ saturation initially again the bands at 549 and 575 nm disappear upon reduction while simultaneously two new signals at 460 and 490 nm arise. The latter were only observed under CO_2_ saturated conditions while the signals emerging above 600 nm upon reduction under N_2_ saturated conditions were not detected. At potentials more negative than −1.8 V the bands at 549 and 575 nm reappeared together with a new feature at 499 nm. Based on the calculated spectra (see Figure 6d), this sequential band appearance can be interpreted as the formation of the 1,4-NH_2_-AQ^2–^ dianionic species with coordinating CO_2_ interaction and single-bound CO_2_, which transforms into a dual CO_2_ adduct at more negative potentials. As presumed from the CV graphs in Figure 4b, no features from the 1,4-NH_2_-AQ^•–^ radical species were observed under CO_2_ saturated conditions in the SEC measurements. Summing up, the calculated results of 1,4-NH_2_-AQ show more pronounced deviations from the experimental spectra as observed in the AQ case and a clear band assignment is more difficult due to overlapping features.

The Δ absorbance curves as well as the absorbance-potential curves for the corresponding absorption bands of 1,4-NH_2_-AQ are summarized in Figure S4.

**Figure 7:**
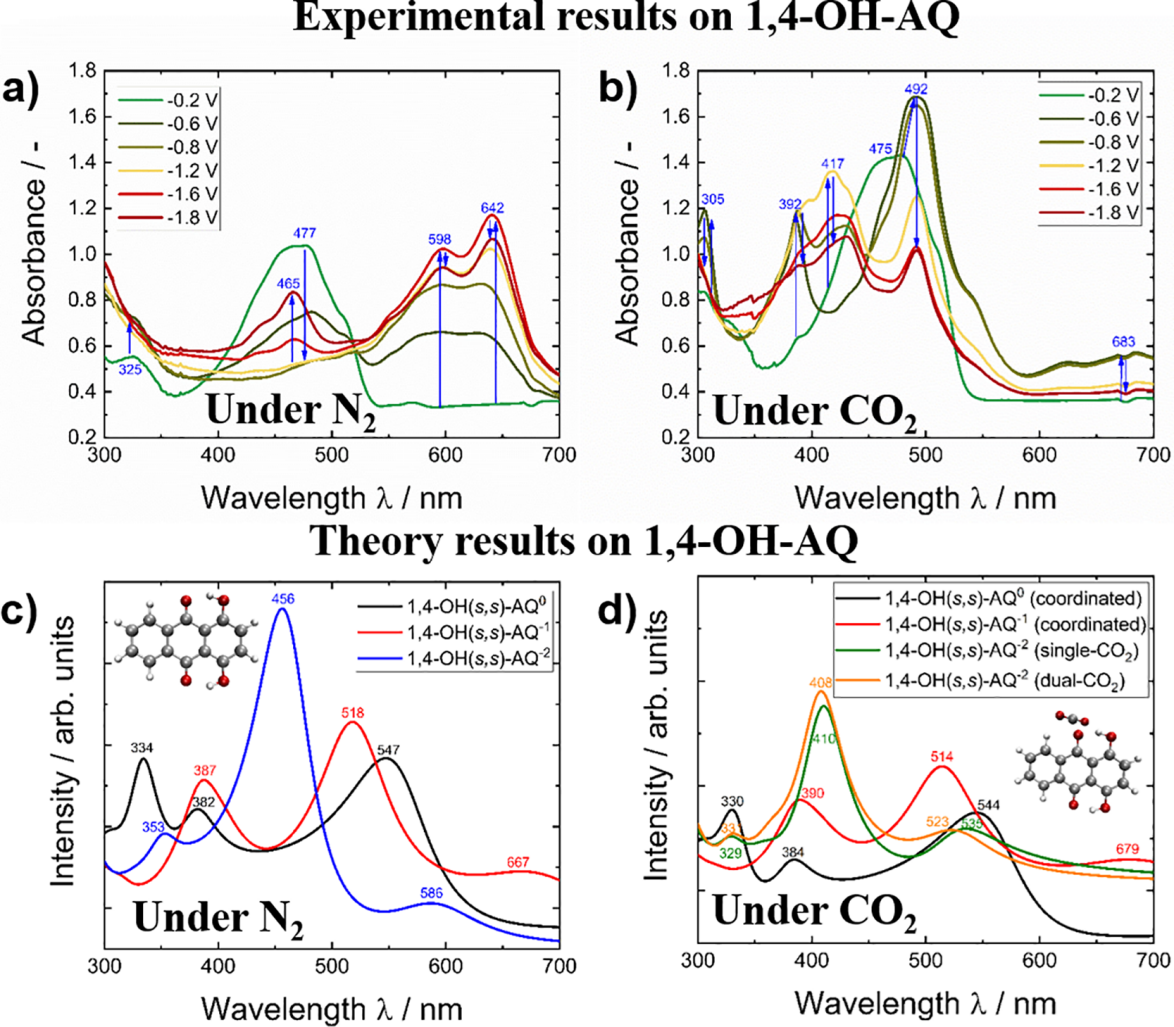
SEC graphs of 1,4-OH-AQ during reduction under (a) N_2_ conditions and (b) CO_2_ saturated conditions. The upper graphs show experimental results, whereas the lower graphs show the DFTB calculated spectra. The insets refer to the geometry optimized structures of 1,4-OH(*s,s*)-AQ (c) and to the coordinated CO_2_:1,4-OH(*s,s*)-AQ^•–^ structure (d).

As reported in the literature,^80^ for the hydroxy-substituted AQ derivatives, conformational isomers in which the hydroxy groups can be aligned with the hydrogen atom pointing toward the carbonyl group (*syn*) or in the opposite direction (*anti*) exist. The conformation of the hydroxy group strongly influences the optical behavior. In the case of 1,4-OH-AQ, all isomers were calculated as depicted in Figure S5, and the (*syn,syn*) configuration was identified as the thermodynamically most stable conformer, which is shown in Figure 7. For both disubstituted materials, the calculated high wavelength transitions were bathochromically shifted compared to the experimental values.

As in the case of 1,4-OH(*s,s*)-AQ, the double reduced species with nonbonded CO_2_ did not converge during the calculations, and no respective spectrum is depicted in Figure 7d. Upon reduction of 1,4-OH-AQ under N_2_ in Figure 7a, the rising bands at 598 and 642 nm can be correlated with the formation of the 1,4-OH-AQ^•–^ radical species. The strongest absorption feature of this species, according to calculations in Figure 7c, should occur at around 518 nm, but is only observed as a weak band in the experimental spectra. At potentials more negative than −1.6 V, the relative intensity of the features above 590 nm slightly diminishes, while a strong signal peak at 465 nm arises, which can be assigned to the formation of the 1,4-OH-AQ^2–^ dianionic species as shown by the good correlation to the calculated peak at 456 nm. Upon reduction in CO_2_ saturation, a completely different behavior compared to the amino species is observed. Already at moderate potentials like −0.6 V, bands centered at 392, 492, and 683 nm arise, which can be identified as 1,4-OH-AQ^•–^ radical species. This observation is in accordance with the nearly identical AQ reduction features observed in the CV curves under N_2_ and CO_2_, shown in Figure 4c. At more negative potentials under CO_2_ saturation, absorption at 417 and 492 nm was observed which correlate to covalently single and dual bound [X-AQ-(CO_2_)_*n*_]^2–^ species, as suggested by the calculated spectra in Figure 7d. Because the calculated spectra for covalent 1,4-OH-AQ-CO_2_ species with one (single) or two bound CO_2_ molecules (dual) show nearly identical theoretical spectra, no clear statement regarding formation of a dual-CO_2_ bound AQ species can be given from the SEC data in Figure 7b. Further discussion of the [X-AQ-(CO_2_)_2_]^2–^ species of 1,4-OH-AQ can be found in section 3.3. Despite the fact of nearly unchanged CV curves in Figure 4c upon CO_2_ addition, the SEC in Figure 7 clearly reveals an interaction between CO_2_ and the reduced 1,4-OH-AQ species. The Δ absorbance curves as well as the absorbance–potential curves for the corresponding absorption peaks of 1,4-OH-AQ are summarized in Figure S6. Summing up, similar to 1,4-NH_2_-AQ shown in Figure 6, 1,4-OH-AQ also shows more pronounced deviations between the experimental and theoretical results, although an overall good agreement is observed. Again, the presence of overlapping features makes a clear band assignment more difficult than in unsubstituted AQ, although it can be stated that (in contrast to AQ and 1,4-NH_2_-AQ), the associated radical species 1,4-OH-AQ^•–^ was also observed under CO_2_ conditions in the SEC measurements.

Although the disubstituted species show the strongest trends for completeness, the SEC results of 1-OH-AQ, 1-NH_2_-AQ, and 1-NH_2_-4-OH-AQ are summarized in Figures S7–S12. The comparison of the SEC data and computed spectra for all investigated compounds shows that only AQ derivatives possessing at least one hydroxy group the presence of a radical species can be observed under CO_2_ saturation.

### 3.3 Influence of the Substituents

The aforementioned impact of CO_2_ on the CV characteristics of differently substituted quinones has become the focus of intense discussions in literature.^25,27,28^ The latest report by Simeon et al. attributes the magnitude of the positive shift of the second-reduction peak observed for various quinone-based compounds under CO_2_ saturated conditions to the Lewis basicity of the substituent groups. According to Simeon et al., quinones displaying a weak binding affinity to CO_2_ show a moderate shift in the second-reduction peak, while for compounds showing high binding affinities, a pronounced shift, sometimes even resulting in one concerted 2-electron-reduction peak, is observed.^28^ It should be mentioned at this point that Simeon et al.^28^ and Tam et al.^27^ were only investigating substituents without any intramolecular hydrogen-bonding properties. In contrast, hydroxy and amino substituents in the β-position were explicitly considered in the present study due to (i) their ability to form intramolecular hydrogen bonds and (ii) their presumed strong interaction with bound CO_2_. Following the CV graphs in Figure 4 and according to the nomenclature used by Simeon et al., the amino groups in 1,4-NH_2_-AQ represent a strongly interacting system that can be recognized via a complete merging of the second-reduction peak with the first one when exposed to CO_2_. In case of 1,4-OH-AQ, the opposite trend is observed, namely unchanged electrochemical features under the influence of CO_2_, as compared to the amino-substituted AQs. The latter finding can be identified as an entirely new class of interaction. In case of all other molecules investigated, namely AQ, 1-OH-AQ, 1-NH_2_-AQ, and 1-NH_2_-4-AQ, a performance behavior in between these two extremes, identified as weak and strong binding by Simeon et al., is observed.

**Figure 8:**
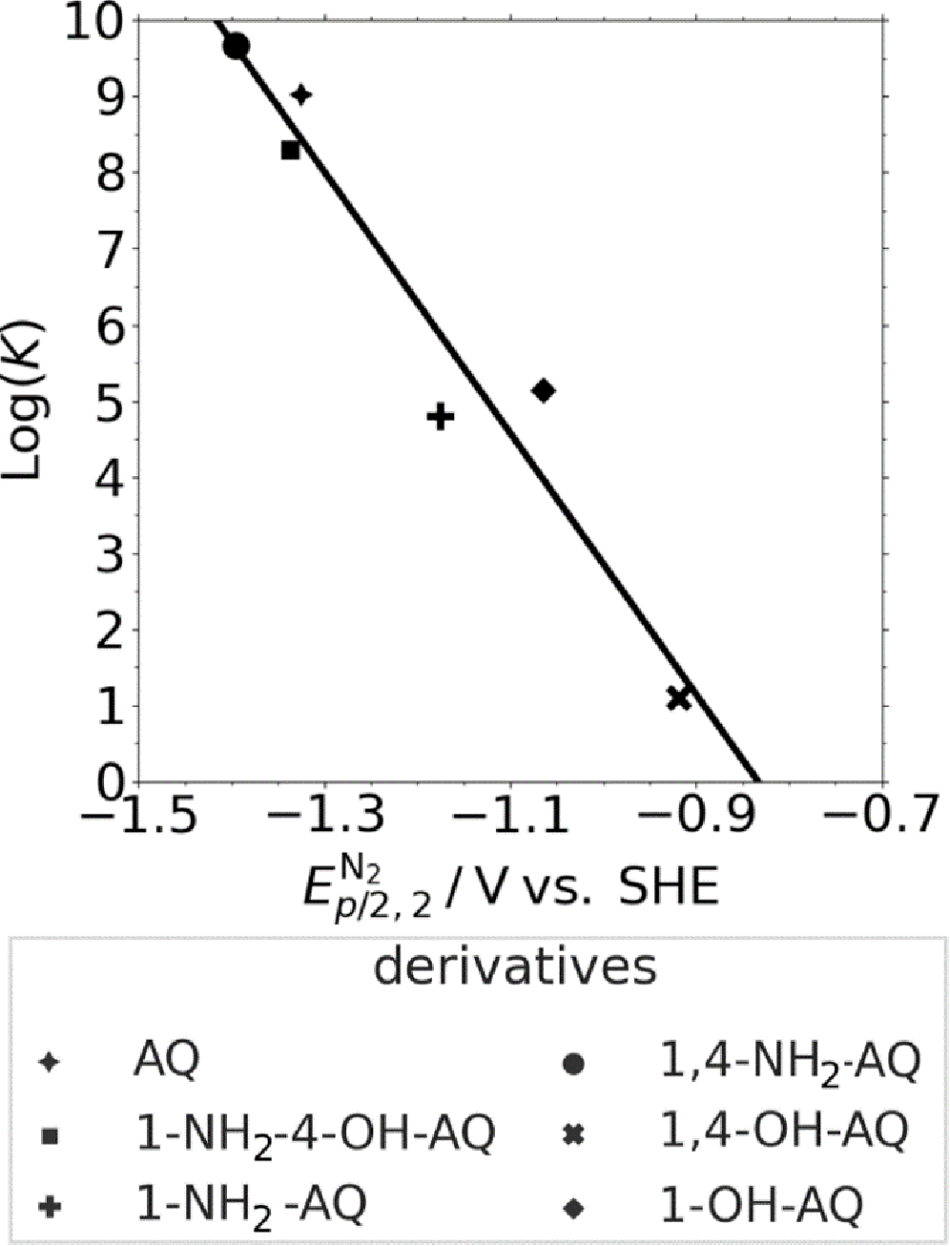
Correlation plot of the log(*K*_b,CO_2__) values versus the electrochemical potential of the second-reduction peak *E*_p/2_ forming X-AQ^2–^ under inert conditions. The correlation value *R*^2^ for the regression shown was determined to be 0.94.

DuBois et al.^19^ and Barlow and Yang^51^ have demonstrated a nearly linear correlation between the logarithmic CO_2_ binding constant log(*K*_b,CO_2__) and the second-reduction under inert conditions *E*_p*,*2_. Figure 8 illustrates this correlation for the investigated systems based on the calculated values listed in Table 1.

As anticipated from literature, these log(*K*_b,CO_2__) values show a quite good correlation with *E*_p*,*2_ determined for the different X-AQ molecules in inert conditions, yielding an *R*^*2*^ of 0.94 (see Figure 8). Even though Nagaoka et al.^18^ used a different polarographic method for the determination of *K*_b,CO_2__, their results for 1,2-OH-AQ and 1,8-OH-AQ are in good agreement with the present results for the OH derivatives. The trend shown in Figure 8 can be understood on the basis that from a qualitative perspective, more negative second-reduction potentials enable a stronger equilibrium interaction of X-AQ^2–^ with CO_2_. However, more negative reduction potentials also imply that a higher energy input is required for the reduction process.

**Figure 9:**
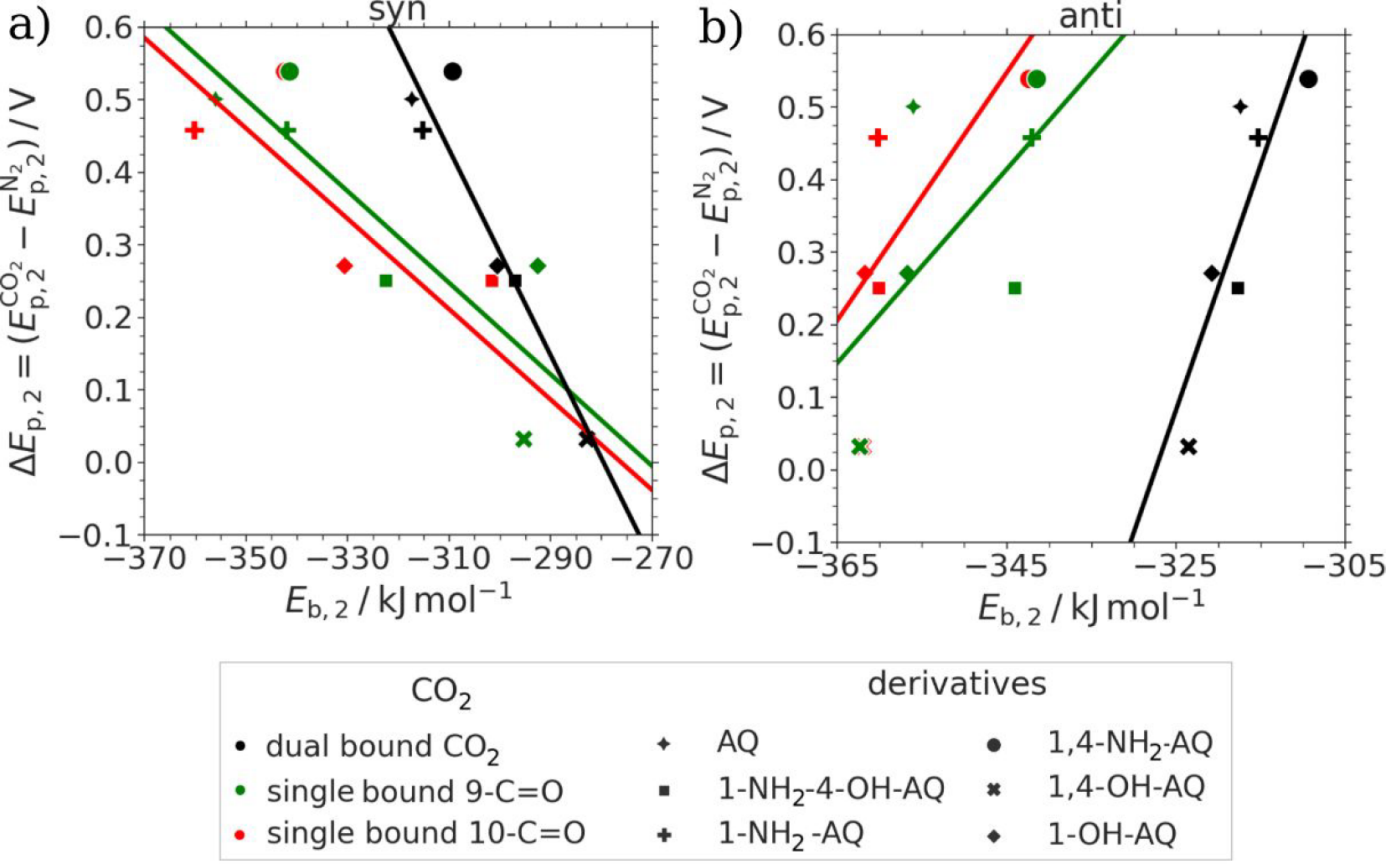
Correlation plots of calculated binding energies with the respective potential shift of the second-reduction peaks Δ*E*_p,2_ versus the binding energies of the covalent CO_2_ and AQ^2–^ species *E*_b,2_ for single and dual CO_2_ adducts of the (a) *syn* confomers and (b) *anti* confomers.

In addition to this purely experimental correlation in Figure 8, the identification of suitable descriptions and explanations for these trends with the help of theoretical calculations was another major target of this study. Figure 9 depicts the correlation of the DFTB calculated binding energy (Δ*E*_b*,*__2_) with the peak potentials of the difference in the second-reduction peaks (Δ*E*_p*,*__2_) calculated via eq 2:
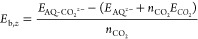
3

Analysis of the binding energies of the covalently bonded [X-AQ-(CO_2_)_*n*_]^2–^ species in Figure 9a,b revealed a different behavior separated into the two different conformers for the hydroxy groups. The first interesting result is that for all materials, the calculated stability of the single-bound adducts is higher compared to the dual-bound species. These thermodynamic considerations are in accordance with our spectroscopic results and present an entirely novel aspect in this research area. Another factor worth noting is that the trend in binding energies is nearly opposite when comparing the *anti* with the *syn* conformers (see Figure 9). As shown within this work by significantly improved *R*^2^ values in Figure 9 and also in the recent work by Gallmetzer et al.,^52^ the *syn* conformation is the most likely occurring one for nearly all cases. Based on this finding, we regard the data shown in Figure 9a as the most reliable description for the stability of the CO_2_ bonded dianions reported to date.

As expected, Figure 9a revealed a significant difference in binding energies *E*_b*,*__2_ for the single-bound CO_2_ molecule with respect to the carbonyl group adjacent to positions 9 or 10. When considering the *E*_b*,*__2_ values for the single-bound species, only a very crude qualitative trend between the binding energy and the difference in the second-reduction peak potentials Δ*E*_p*,*__2_ is observed. When considering the dual-bound species, the same trend is observed with a more quantitative correlation with an improved *R*^2^ value in the range from 0.72 to 0.90 from *anti* to *syn*. The unsubstituted AQ and amino-substituted dianions were determined to have significantly higher binding energies in comparison to the (dual) hydroxy derivatives.

These results from Figure 9a reveal that the stabilities of the [X-AQ-(CO_2_)_2_]^2–^ species show a good correlation with the potential differences in the second-reduction peaks. In addition, a similar correlation plot of the log(*K*_b,CO_2__) values from Table 1 versus the computed binding energies *E*_b*,*2_ for the single-bound and dual-bonded CO_2_ is depicted in the Figure S13. For the single-bound CO_2_ adducts, a moderate correlation similar to Figure 9 is observed. However, the computed *E*_b*,*2_ values for the *syn*-[X-AQ-(CO_2_)_2_]^2–^ species versus the log(*K*_b,CO_2__) values show an excellent correlation with an *R*^2^ value of 0.90, indicating that the theoretically determined binding energy is a highly suitable descriptor to probe the CO_2_ capture capability of the investigated X-AQ compounds. It was shown in the recent work by Gallmetzer et al.^52^ that the theoretically determined electrochemical potentials also correlate very well with experimental reference data in the majority of cases. Since both the AQ-CO_2_ interaction potential as well as the electrochemical potentials depend strongly on the accurate description of the electronic structure of the target compounds, future studies will aim to identify which aspect of the theoretical calculation is the key driver for the strong correlations observed.

**Figure 10:**
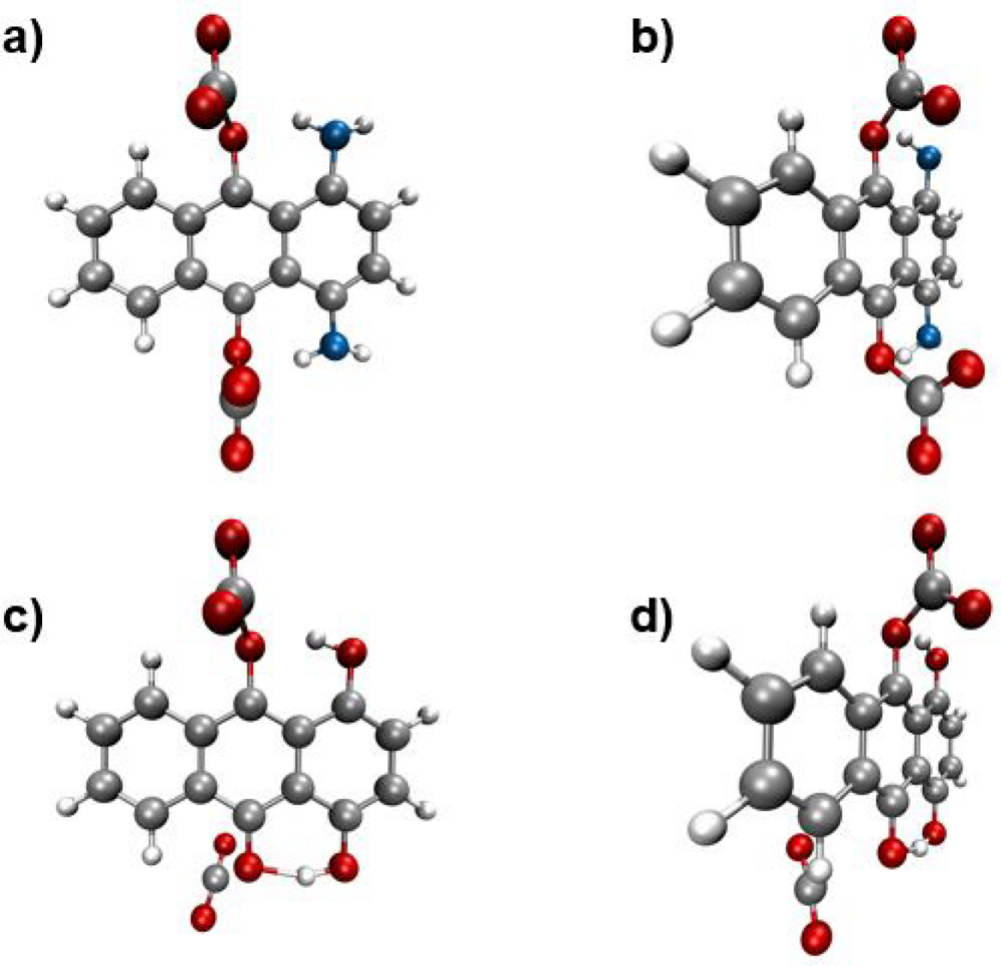
Illustration of the optimized geometries in case of two CO_2_ molecules covalently bound to (a, b) 1,4-NH_2_-AQ and to (c d) 1,4-OH-AQ. The left graphs (a, c) are the top view, while the right graphs (b, d) refer to a 90° rotated side view.

The calculations even revealed that dual-bound species of 1-OH-AQ, 1,4-OH-AQ, and 1-NH_2_-4-OH-AQ partially dissociated during the optimization steps, which is schematically depicted in Figure 10.

Figure 10a,b shows the calculated, optimized structures of two CO_2_ molecules bound to 1,4-NH_2_-AQ from top view and side view. In accordance with the results discussed in section 3.2, the [1,4-OH-AQ-(CO_2_)_2_]^2–^ structure was determined to partially dissociate due to lack of the stability from a thermodynamic point of view, as depicted in Figure 10c,d.

Up to this point, the discussion was restricted to the well-described interaction behavior of the X-AQ^2–^ species with CO_2_. However, some experimental observations, like the unchanged CV behavior of 1,4-OH-AQ under CO_2_, could not be fully understood by means of the dianionic species. In addition to the DFTB calculation results of the radical-CO_2_ species, which are shown in the following, the SEC data presented in this work also show significant changes in the moderate potential range. The findings are in agreement with DuBois et al.^19^ and Tam et al.,^27^ who also described changes in the UV–Vis spectra of the radical species upon presence of CO_2_ exposure.

When considering the perspective of a chemical equilibrium, the shift of the second-reduction peak under CO_2_ can be understood as a combination of the stability of a CO_2_:X-AQ^•–^ radical and the [X-AQ-(CO_2_)_2_]^2−^ species. In the case of a “strong binding” found for 1,4-NH_2_-AQ, the radical species is apparently less stable in the presence of CO_2_ compared to the corresponding dianion, which in turn results in the observed concerted 2-electron-reduction peak (Figure 4b). On the other hand, the unchanged CV features of 1,4-OH-AQ under N_2_ and CO_2_ atmosphere (Figure 4c) are an indication of a similar or even increased, high stability of the respective radical species which forms under both conditions. This observation was also reported by Nagaoka et al.;^18^ however, at that time the authors could not provide further insights into the origin of these unchanged CV characteristics.

**Figure 11:**
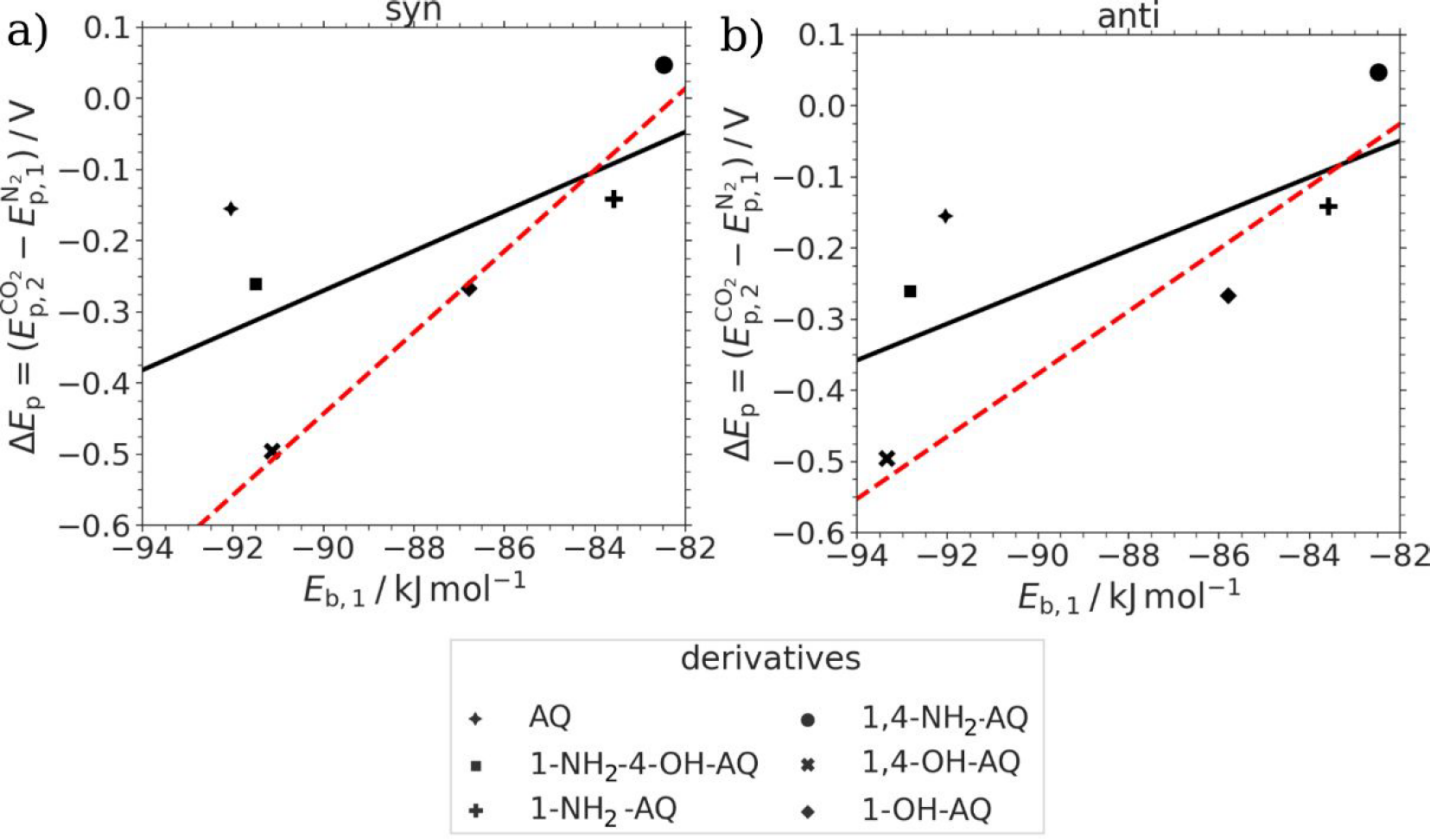
Correlation plots of the potential difference of the second-reduction peak under CO_2_ and the first-reduction peak under N_2_, Δ*E*_p,2–1_ versus the estimated binding energy of the coordinated CO_2_:X-AQ^•–^ species *E*_b,1_ of the (a) *syn* confomers and (b) *anti* confomers. The black lines show the trend including all six derivatives, whereas the red, dotted line excludes AQ and 1-NH_2_-4-OH-AQ.

According to this theory, the stability of the X-AQ^•–^ radical species under interaction with CO_2_ should correlate with the potential difference between the second-reduction peak under CO_2_ and the first-reduction peak under inert N_2_ conditions. In order to confirm this, binding energies of CO_2_ in the CO_2_:X-AQ^•–^ species, *E*_b,1_, were computationally calculated for the six investigated AQ derivatives and related with the experimentally observed potential differences of the reduction peaks under CO_2_ (second reduction) and N_2_ (first reduction) Δ*E*_p,2–1_ as shown in Figure 11.

The binding energies *E*_b*,*__1_ used for the correlation in Figure 11 were calculated from the corresponding DFTB calculations (according to eq 3), and the potential differences were determined from the CV graphs depicted in Figure 4 (see also Table S1) according to the following formula in eq 4:
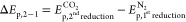
4

Although the potential difference Δ*E*_p,2–1_ in eq 4 appears to be very similar to Δ*E*_p*,*__2_ in eq 2, we propose that the shift of the second-reduction peak under CO_2_ toward the first peak under N_2_ represents a more suitable experimental quantity to represent the radical stability compared to its Δ*E*_p*,*__2_ counterpart (eq 2).

When excluding 1-NH_2_-4-OH-AQ and AQ, the comparison in Figure 11a reveals a clear correlation (red line) between the stability of the radical species in CO_2_ saturated conditions and the shift of the second-reduction peak under CO_2_ toward the first-reduction peak under N_2_. One possible reason for the observed deviation in the stability of AQ is the lack of any steric hindrance upon CO_2_ binding, which was also recently suggested by Bui et al.^33^ Furthermore, it has to be stated that additional research is required to provide a comprehensive understanding of the stabilities of CO_2_:X-AQ^•–^ species, since the experimentally determined value of *ΔE*_p,2–1_ still consists of equilibrium contributions of both the radical as well as the final X-AQ^2–^ species. Another possibility for the observed deviating behavior of certain derivatives is that the type of electrochemical and/or chemical mechanism might also be dependent on the actual substitutions of the AQ lead structure.

In general, more stable CO_2_:X-AQ^•–^ species involving intramolecular hydrogen bonding of hydroxy groups show less affected CV graphs when changing from N_2_ to CO_2_ saturated conditions as compared to amino-substituted AQs, which show a concerted 2-electron reduction under CO_2_. The CV of 1,4-OH-AQ in Figure 4c is exemplary for this trend, as no effect of CO_2_ on the individual peaks was observed, although the calculation results and SEC data shown in Figure 7 clearly prove the formation of CO_2_:quinone adducts. However, 1-NH_2_-4-OH-AQ displays a behavior that is notably deviating from the observed trend, although the thermodynamically more stable *syn* conformer is the one closer to the linear regression. A possible explanation for the peculiar behavior of AQ and 1-NH_2_-4-OH-AQ is that their reduction potential of around −0.66 V is at an optimum energetic value for CO_2_ capture, as pointed by Bui et al.^33^ Despite the above-mentioned differences, this work represents, to the best of our knowledge, the first instance in which the binding energies of CO_2_ and X-AQ^•–^ radical species are reported. They are found to be qualitatively in good agreement with the experimental observations of radical species measured by SEC, as given in section 3.2. For this computational consideration, the starting geometry of CO_2_ and the AQ derivatives was selected to be close to the substituent group (Figure S14). However, it should be stated that for further in-depth studies, the other three possible CO_2_ approaching sites will also be considered, especially as the radical is not as localized on the carbonyl groups as is the case for the dianionic species.

## 4 Conclusions

In this study, the electrochemical and spectroelectrochemical behavior of AQ derivatives substituted with amino and hydroxy groups adjacent to the carbonyl moieties were thoroughly investigated. Upon electrochemical reduction under CO_2_, saturated conditions of all molecules except 1,4-OH-AQ show a shift of the second toward the first-reduction peak when compared to the reduction behavior under inert (N_2_) saturated conditions. In addition to seminal studies, we thereby have identified the hydroxy-substituted AQs as a special case displaying an even weaker CO_2_ binding affinity, as the term “weak bonding” commonly employed in the literature suggests. This weak bonding was further characterized by evaluating the associated CO_2_ binding constants *K*_b,CO_2__ as well as computational calculations of the associated binding energies, which both suggest molecular dissociation to occur. To the best of our knowledge, this is the first report that quantitatively investigates the impact of AQ substituents involving intramolecular hydrogen bonds, which represents a significant step beyond the previous consideration of only mesomeric/nucleophilicity effects. For all derivatives investigated in this study, a significant change in the UV–Vis absorption spectra indicates the formation of new species via interaction with CO_2_ even in cases in which the CV measurements did not show significant changes. Only in the case of derivatives carrying at least one hydroxy group, absorption bands of X-AQ^•–^ were detectable in SEC under CO_2_ saturated conditions.

In order to complement the experimental measurements, extensive DFTB calculations in the presence and absence of CO_2_ have been performed for the first time within the context of electrochemical CO_2_ capture. The DFTB calculated spectra under inert conditions enable a detailed correlation of the spectral changes upon electrochemical reduction under N_2_. Although the calculated UV–Vis spectra show some minor deviations from the experimental reference, good agreement between theory and experiment was observed in case of the reduced X-AQ species. Furthermore, the spectral changes under CO_2_ saturation can be explained by the formation of different coordinatively and covalently bound CO_2_ species. However, as seen from the measured data, a mixture of several AQ-CO_2_ species according to the reaction scheme displayed in Figure 1 is present at our experimental conditions. Based on the binding energies of these X-AQ-CO_2_ species, it could be deduced that hydroxy substituents stabilize the CO_2_-radical species, while amino substituents display destabilizing properties. Moreover, it was found that the stability of the [X-AQ-(CO_2_)_2_]^2–^ species follows the opposite trend, implying that the presence of amino groups and no substituents demonstrates a stabilizing effect, while for 1,4-OH-AQ, the theoretical calculations even suggested thermodynamic instability and a dissociation of this particular adduct. To the best of our knowledge, this is the first time that not only dianionic species with CO_2_ are described but also an estimation of the contribution of CO_2_:X-AQ^•–^ species was carried out to provide insights into the associated electrochemical CO_2_ capture mechanism. However, we emphasize at this point that the presented results indicate a rough and qualitatively but at the same time highly promising and virtually unexplored field of CO_2_:X-AQ^•–^ species for which precise experimental quantities for X-AQ^•–^ species have to be developed.

Based on the experimental and theoretical results presented in this study, we refrain from the widely proposed mechanism that the electrochemical CO_2_ capture assumed to always result in an [X-AQ-(CO_2_)_2_]^2–^ species has to be adapted, considering instead a less-defined mixture of reduced species as reaction product. Furthermore, it is proposed that changes in the substitution of AQs via hydrogen-bonding groups like amino and hydroxy can selectively stabilize either the radical or dianionic species, which is a crucial aspect for future studies of efficient, quinone-based electrochemical CO_2_ capture strategies. In addition to modifying the binding strengths and reduction potentials of the AQ lead structure, the very recent work by Barlow and Yang^51^ and their suggestion of tuning with hydrogen-donating additives paves the way for an entirely new research direction for electrochemical CO_2_ capture.

## Supporting Information Available

UV–Vis and PL spectroscopic data (Figure S1), blank GC CV data (Figure S2); absorbance vs potential plots and ΔAbsorbance plots for AQ, 1,4-NH_2_-AQ and 1,4-OH-AQ (Figures S3, S4, and S6); computed UV–Vis spectra of the three conformational isomers of 1,4-OH-AQ (*syn*/*syn*; *anti*/*syn*, and *anti*/*anti*) (Figure S5); full SEC graphs and calculated spectra for the reduced species of 1-NH_2_-AQ, 1-OH-AQ, and 1-NH_2_-4-OH-AQ including absorbance vs potential plots and Δabsorbance plots (Figures S7–S12); correlation plot of log(*K*_b,CO_2__) values vs calculated binding energies *E*_b,2_ (Figure S13); and illustration of possible binding sites for CO_2_ (Figure S14) (PDF)
