# Supplementary material for: Direct Electrochemical CO2 Capture Using Substituted Anthraquinones in Homogeneous Solutions: A Joint Experimental and Theoretical Study
Source: J Phys Chem C Nanomater Interfaces. 2022 Aug 15;126(33):14138–54. doi: 10.1021/acs.jpcc.2c03129 (PMC9421899; doi:10.1021/acs.jpcc.2c03129)
Supplement: Supplementary file 1 — jp2c03129_si_001.pdf [file jp2c03129_si_001.pdf]

Direct Electrochemical CO_2 Capture using Substituted Anthraquinones in Homogeneous Solutions – a Joint Experimental and Theoretical Study

Corina Schimanofsky[a], Dominik Wielend[a], Stefanie Kröll[b], Sabine Lerch[b], Daniel Werner[c], Josef M. Gallmetzer[b] , Felix Mayr[a,d], Helmut Neugebauer[a], Mihai Irimia-Vladu[a], Engelbert Portenkirchner[c], Thomas S. Hofer*[b], Niyazi Serdar Sariciftci[a]*

[a] Linz Institute for Organic Solar Cells (LIOS), Institute of Physical Chemistry, Johannes Kepler University Linz, Altenberger Straße 69, 4040 Linz, Austria.

[b] Theoretical Chemistry Division, Institute for General, Inorganic and Theoretical Chemistry, University of Innsbruck, Innrain 80-82, 6020 Innsbruck, Austria

[c] Institute of Physical Chemistry, University of Innsbruck, Innrain 52c, 6020 Innsbruck, Austria

[d] Institute of Applied Physics, Johannes Kepler University Linz, Altenberger Straße 69, 4040 Linz, Austria.

(* Main corresponding author: dominik.wielend@jku.at

Corresponding author theory: t.hofer@uibk.ac.at)

Supporting Information

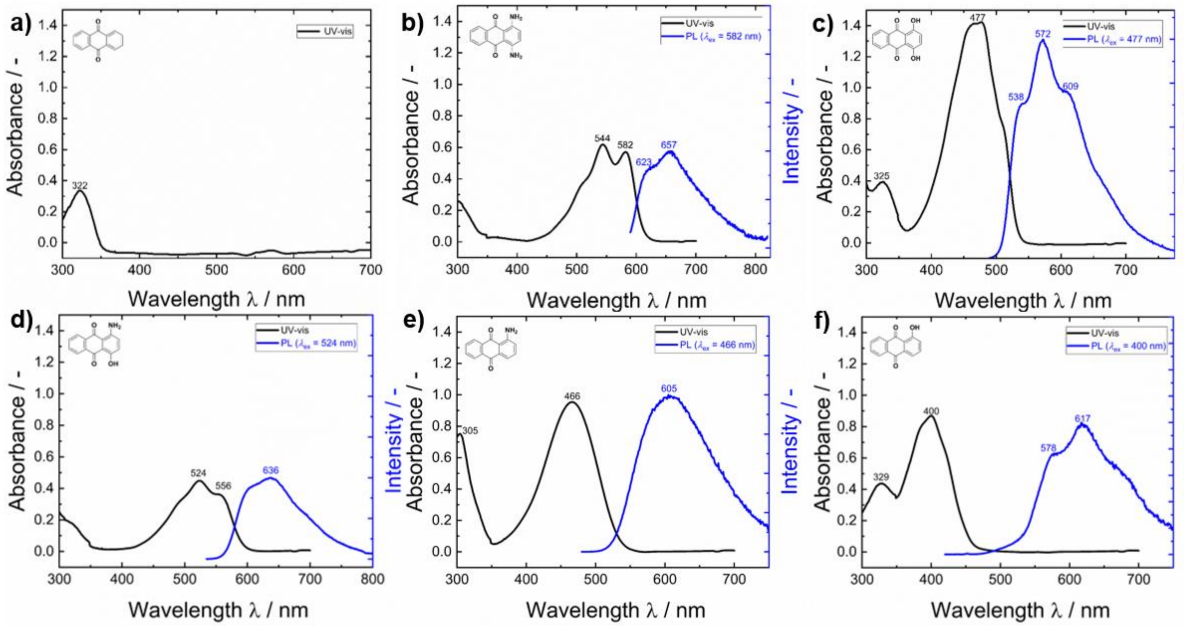

Figure S1: UV-Vis absorption spectra and PL spectra of a) anthraquinone (AQ), b) 1,4-NH_2-AQ, c) 1,4-OH-AQ, d) 1-NH_2-4-OH-AQ, e) 1-NH_2-AQ and f) 1-OH-AQ in MeCN solution. Emission spectra were recorded at the excitation wavelength given, which corresponds to an absorption maximum.

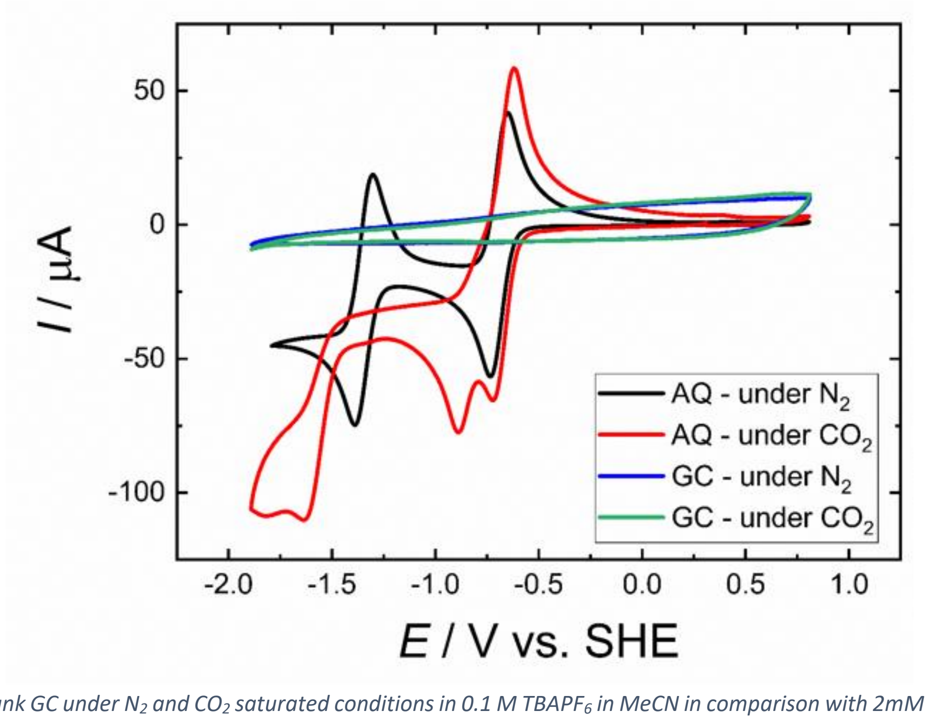

Figure S2: CV of blank GC under N_2 and CO_2 saturated conditions in 0.1 M $TBAPF_6$ in MeCN in comparison with 2mM AQ solution under N_2 and CO_2 saturated conditions. In case of the N_2 saturated conditions, the 2nd cycle is shown whereas in case of CO_2 saturated conditions the 20th cycle is shown.

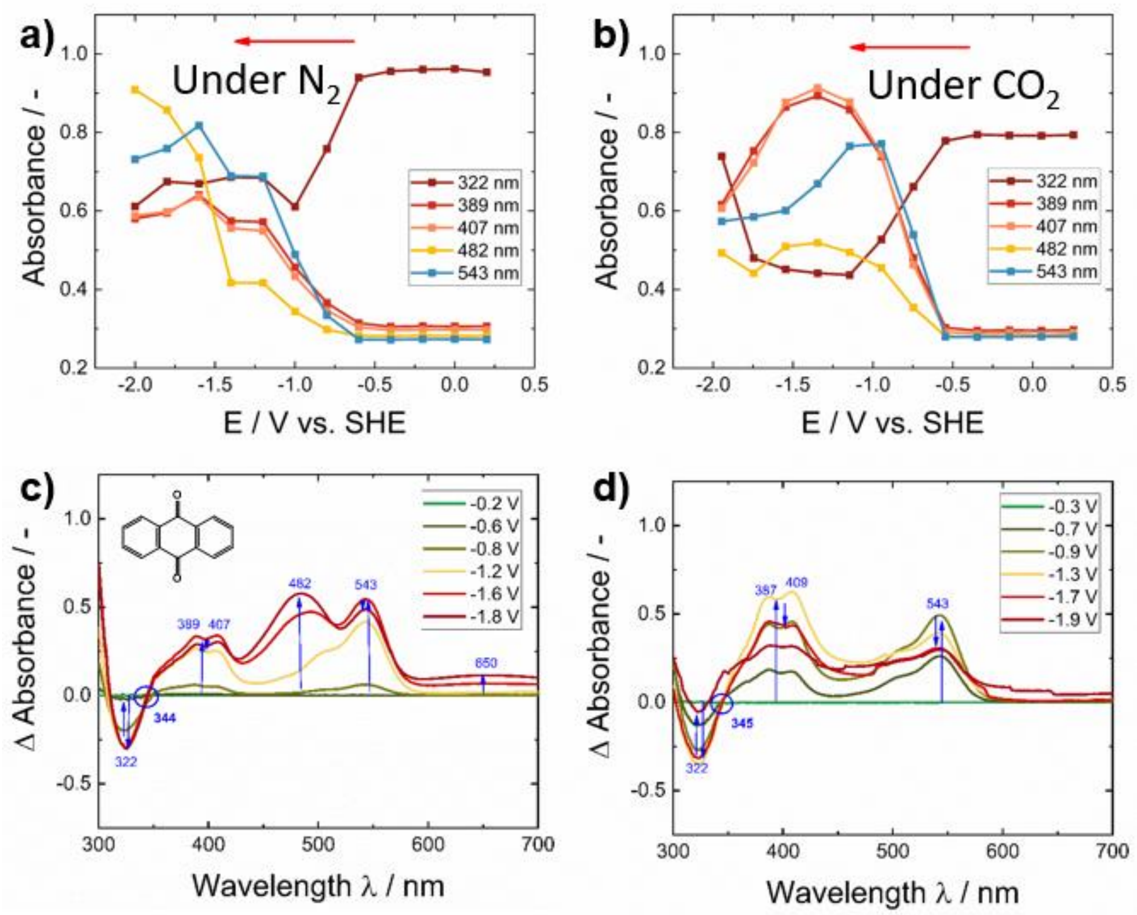

Figure S3: Absorbance-potential curves at the characteristic wavelengths of AQ in a) N_2 saturated conditions and b) CO_2 saturated conditions and delta absorbance curves in c) N_2 saturated conditions and d) CO_2 saturated conditions.

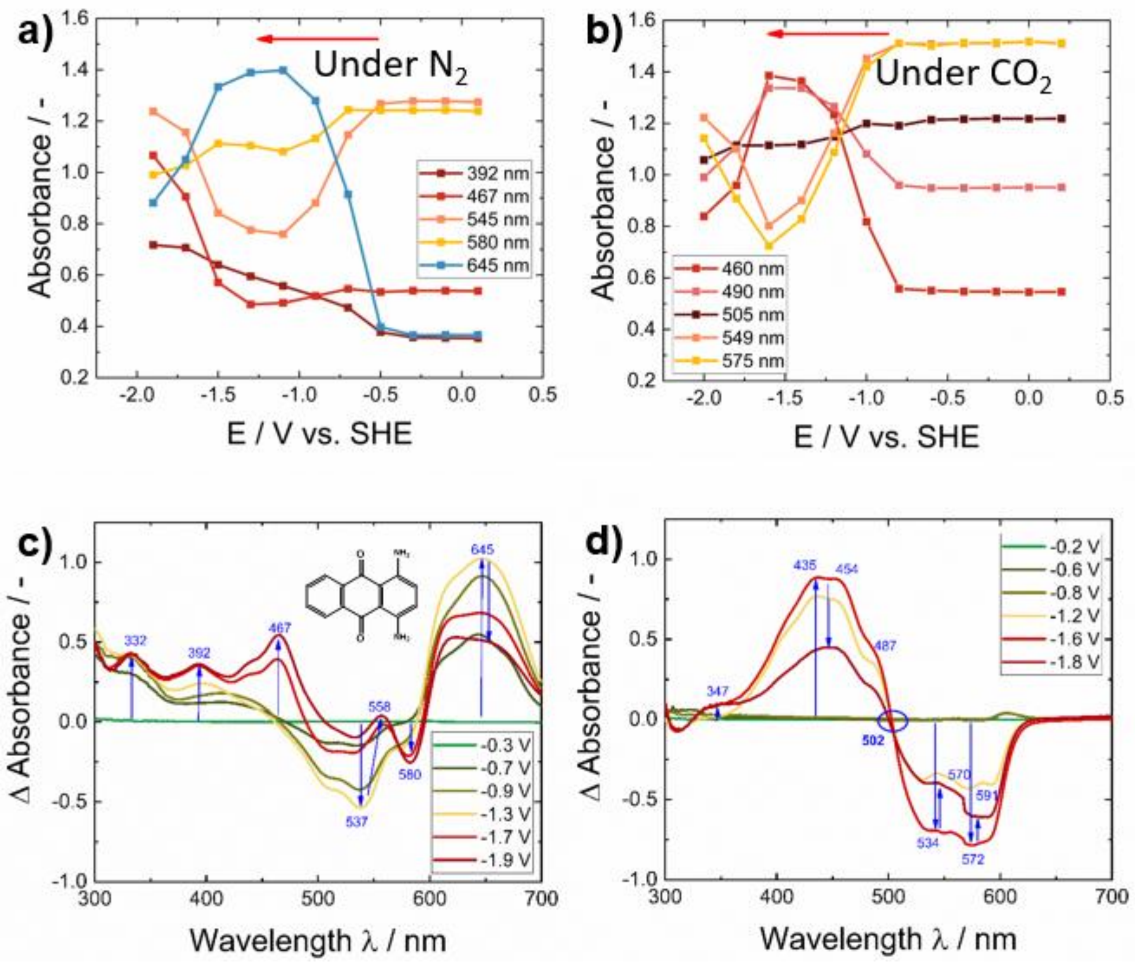

Figure S4: Absorbance-potential curves at the characteristic wavelengths of 1,4-NH_2-AQ in a) N_2 saturated conditions and b) CO_2 saturated conditions and delta absorbance curves in c) N_2 saturated conditions and d) CO_2 saturated conditions.

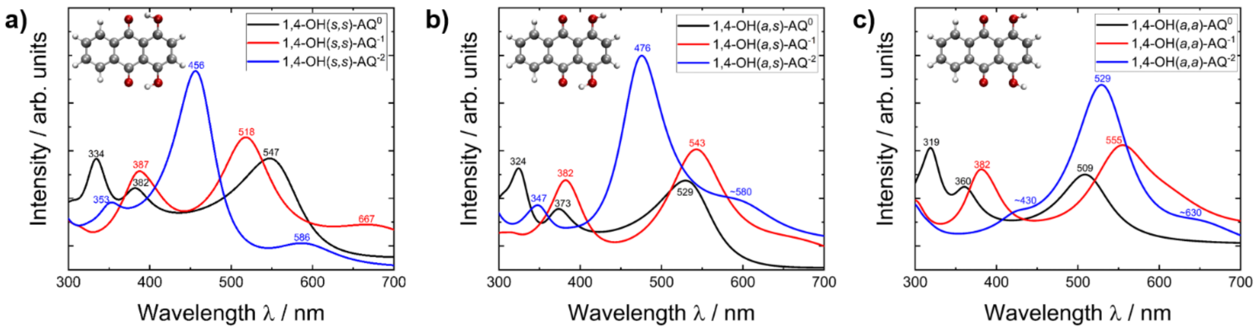

Figure S5: Calculated UV-Vis absorption spectra of 1,4-OH-AQ for a) (syn,syn), b) (anti,syn) and c) (anti,anti) conformational isomers.

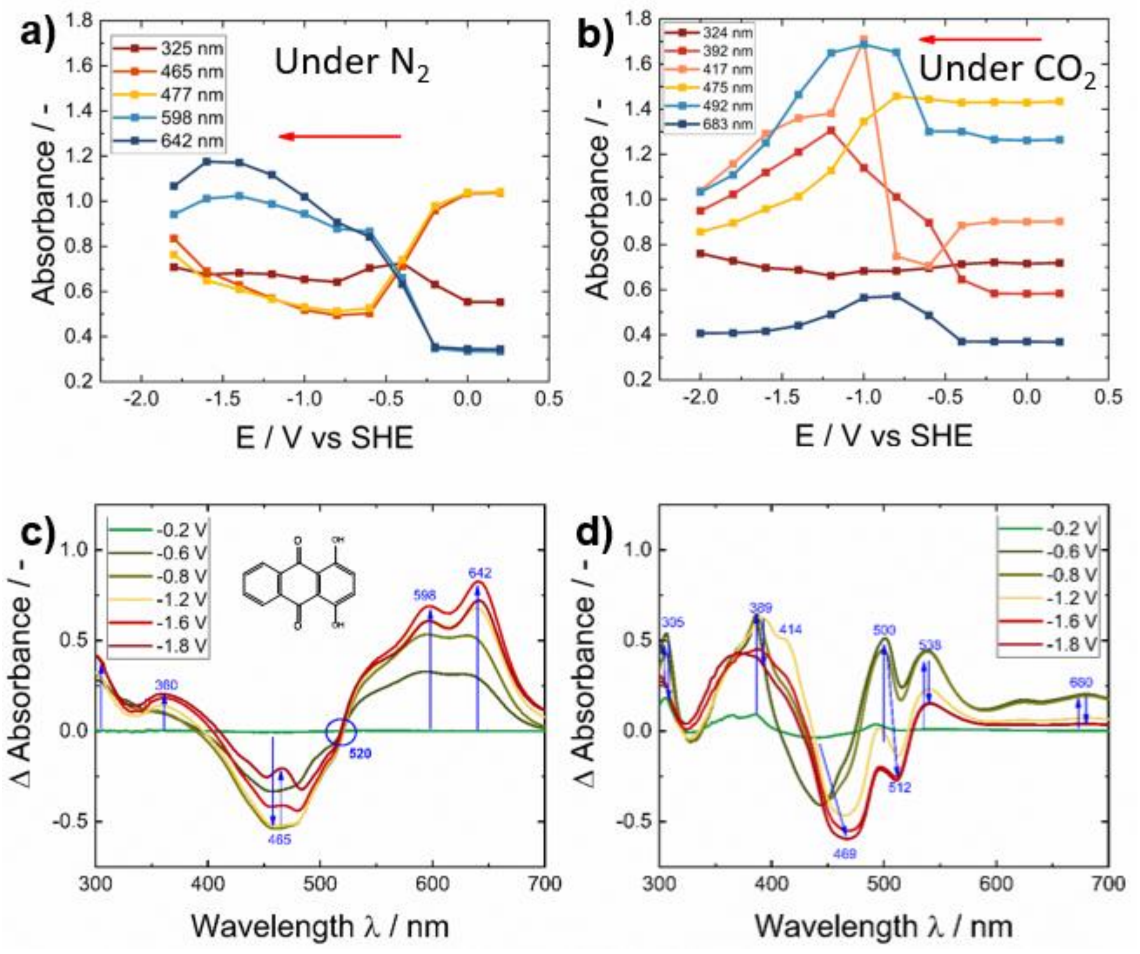

Figure S6: Absorbance-potential curves at the characteristic wavelengths of 1,4-OH-AQ in a) N_2 saturated conditions and b) CO_2 saturated conditions and delta absorbance curves in c) N_2 saturated conditions and d) CO_2 saturated conditions.

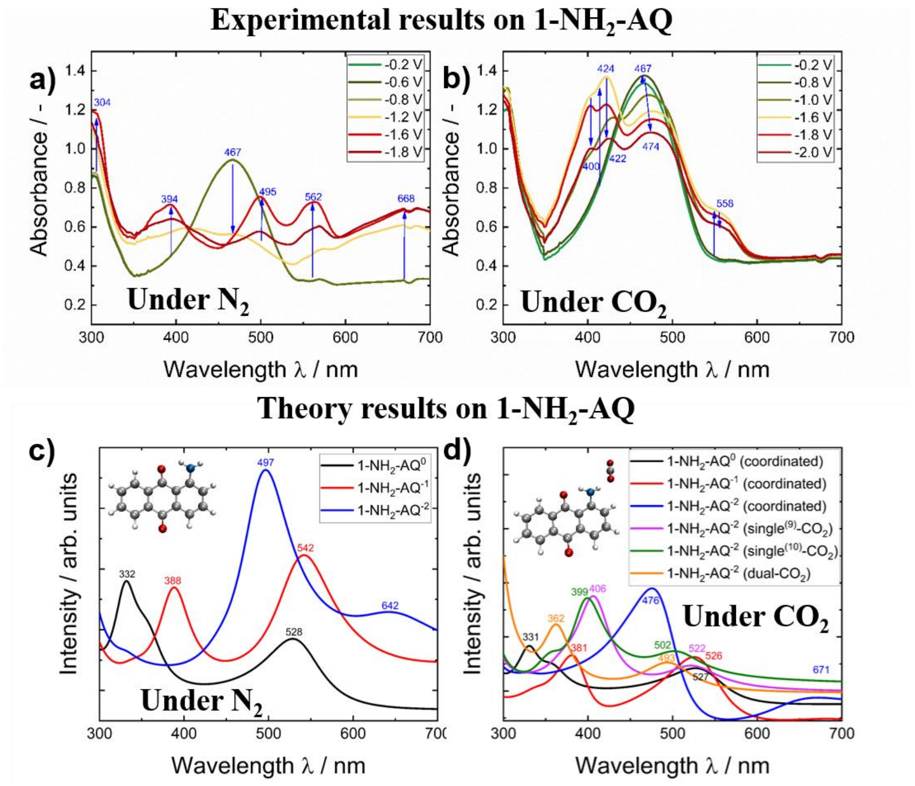

Figure S7: Spectroelectrochemistry graphs of a) 1-NH$_2$-AQ under N$_2$ conditions and b) under CO$_2$ saturated conditions. The upper graphs show experimental results whereas the lower graphs (c & d) show DFTB calculated spectra. The insets refer to the geometry optimized structures of 1-NH$_2$-AQ (c) and to the coordinated CO$_2$:1-NH$_2$-AQ$^{\bullet-}$ structure (d).

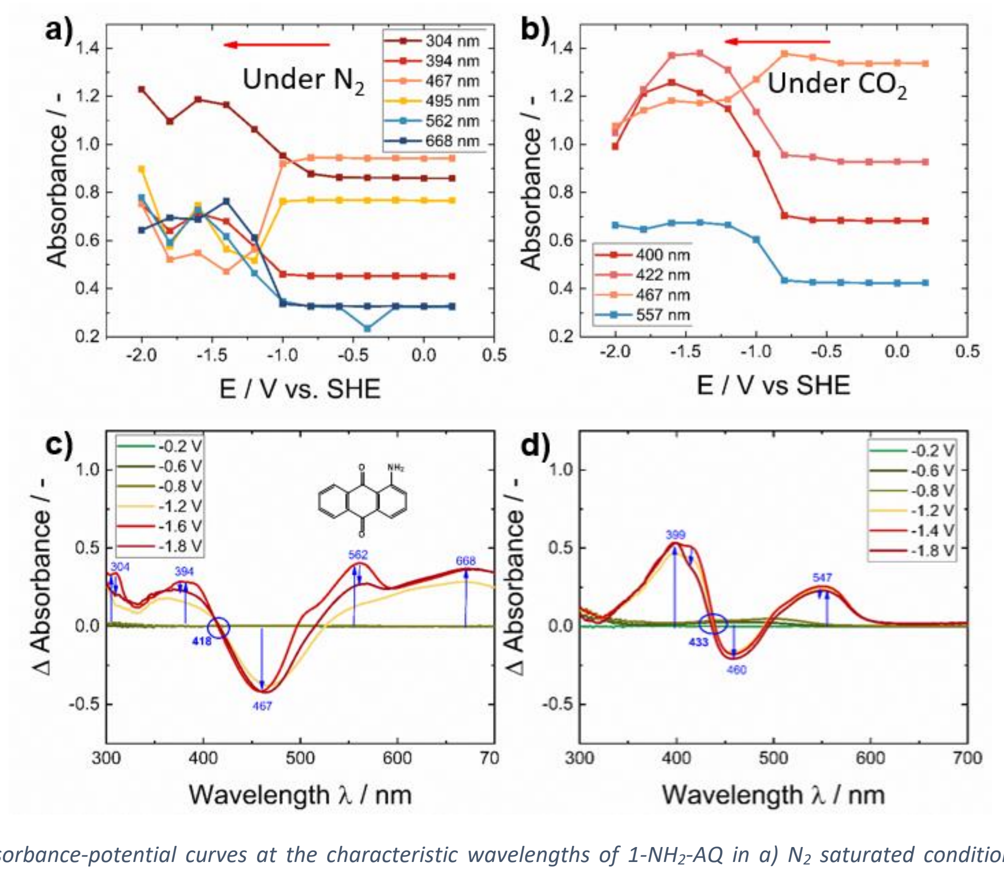

Figure S8: Absorbance-potential curves at the characteristic wavelengths of 1-NH$_2$-AQ in a) N$_2$ saturated conditions and b) CO$_2$ saturated conditions and delta absorbance curves in c) N$_2$ saturated conditions and d) CO$_2$ saturated conditions.

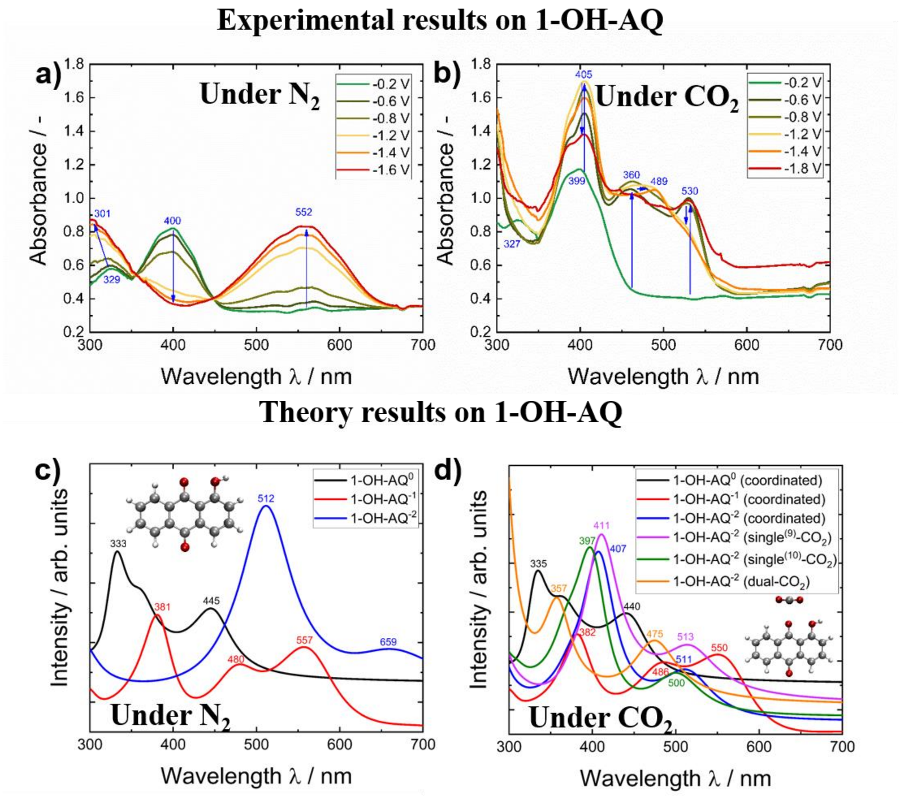

Figure S9: Spectroelectrochemistry graphs of a) 1-OH-AQ under N_2 conditions and b) under CO_2 saturated conditions. The upper graphs show experimental results whereas the lower graphs (c & d) show DFTB calculated spectra. The insets refer to the geometry optimized structures of 1-OH(a)-AQ (c) and to the coordinated CO_2:1-OH(a)-AQ$^{\bullet-}$ structure (d).

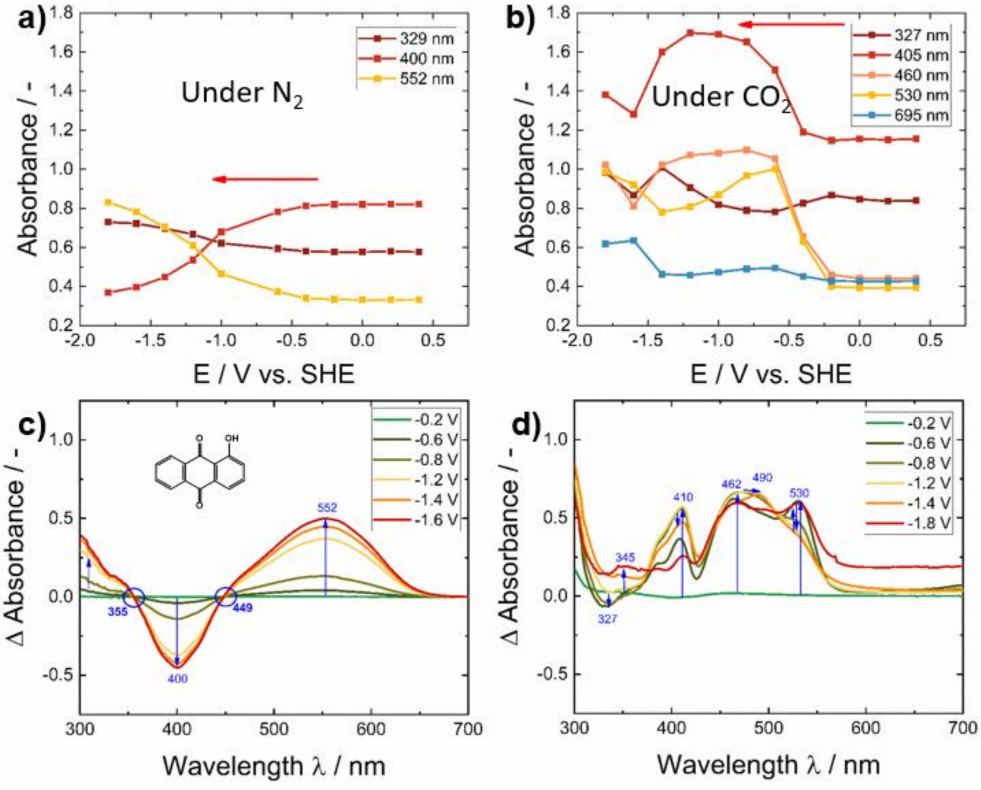

Figure S10: Absorbance-potential curves at the characteristic wavelengths of 1-OH-AQ in a) N_2 saturated conditions and b) CO_2 saturated conditions and delta absorbance curves in c) N_2 saturated conditions and d) CO_2 saturated conditions.

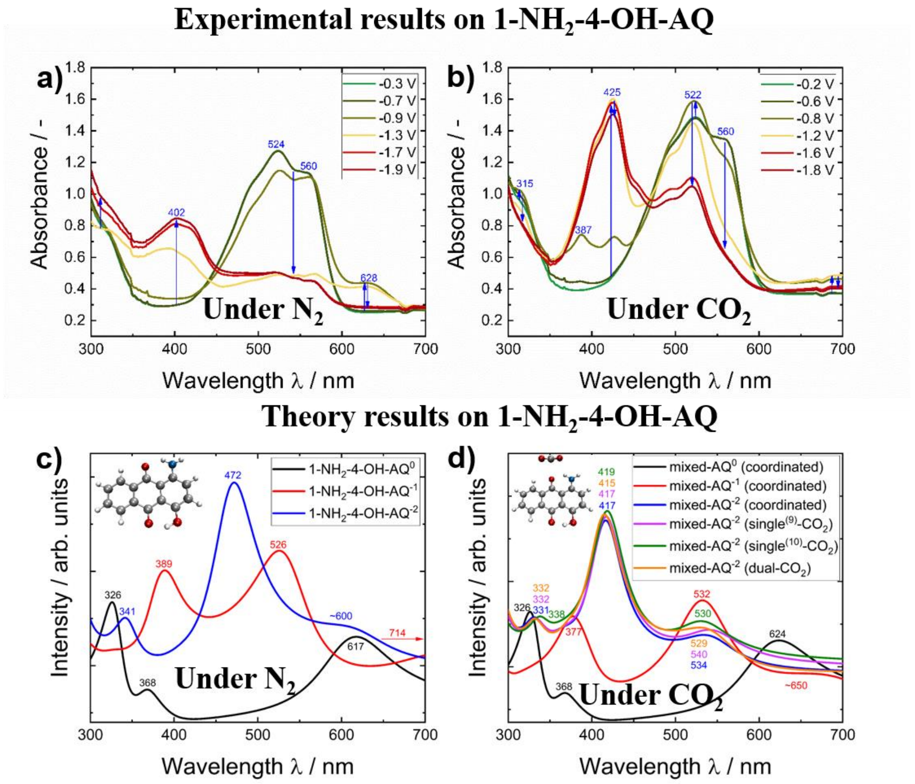

Figure S11: Spectroelectrochemistry graphs of a) 1-NH_2-4-OH-AQ under N_2 conditions and b) under CO_2 saturated conditions. The upper graphs show experimental results whereas the lower graphs (c & d) show DFTB calculated spectra. The insets refer to the geometry optimized structures of 1-NH_2-4-OH(s)-AQ (c) and to the coordinated CO_2:1-NH_2-4-OH(s)-$AQ^{\bullet -}$ structure (d).

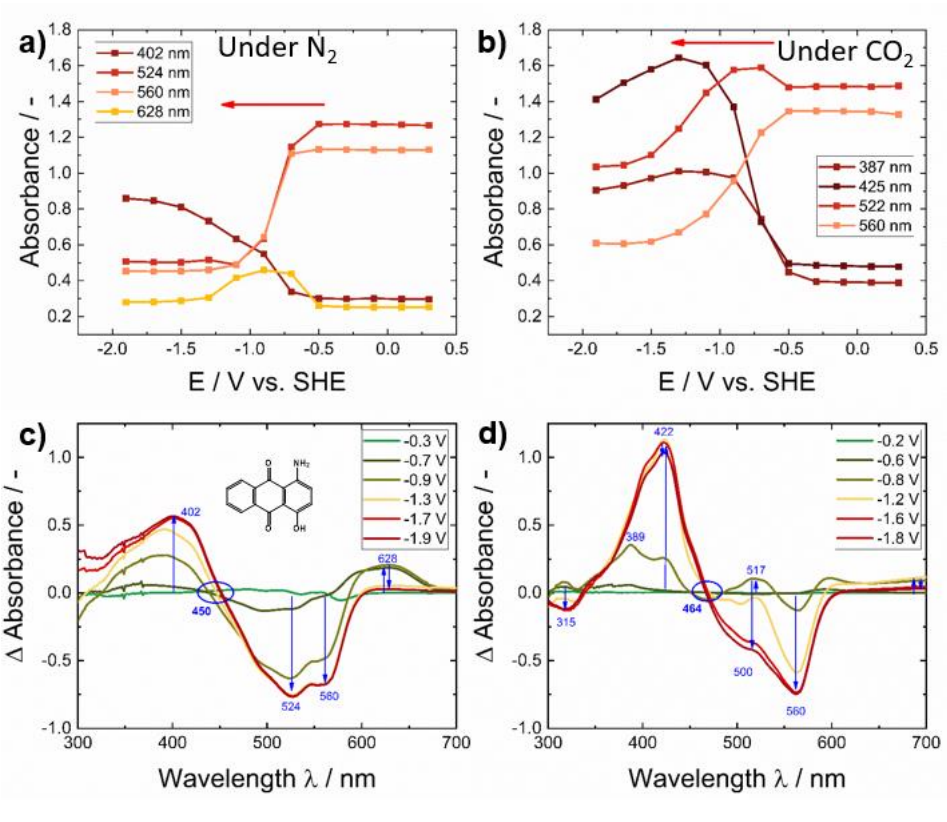

Figure S12: Absorbance-potential curves at the characteristic wavelengths of 1-NH_2-4-OH-AQ in a) N_2 saturated conditions and b) CO_2 saturated conditions and delta absorbance curves in c) N_2 saturated conditions and d) CO_2 saturated conditions.

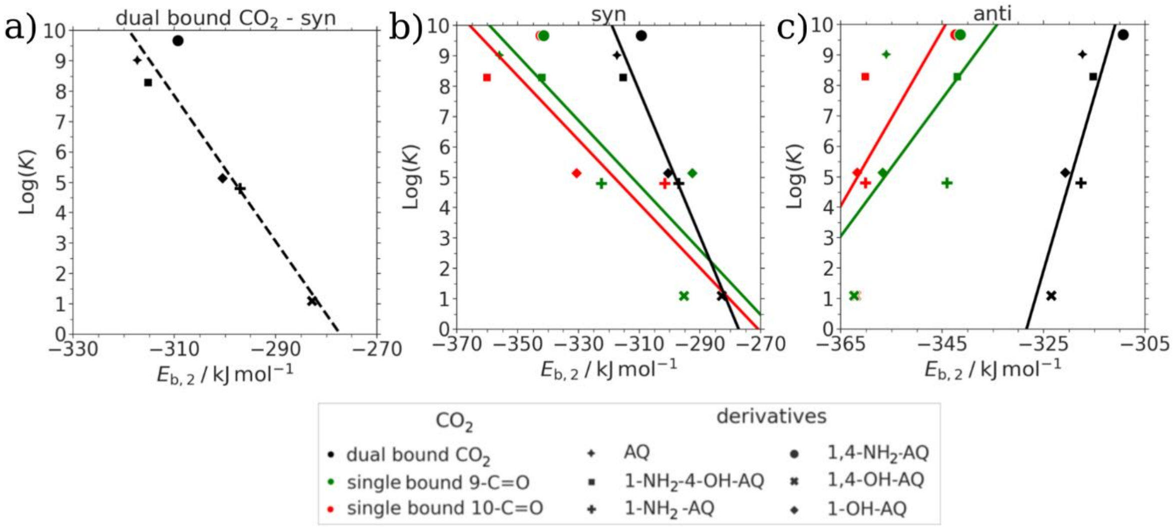

Figure S13: Correlation plots of a) calculated binding energies of the covalent dual bound CO_2 and AQ^{2-} adduct species $E_{b,2}$ versus the log($K_{b,CO2}$) values and correlation plots of the binding energies of the covalent CO_2 and AQ^{2-} species $E_{b,2}$ for single and dual CO_2 adducts of the b) syn confomers and c) anti confomers versus the log($K_{b,CO2}$) values.

Table S1: Summary of the peak potentials E_p of the first and the second reduction peak under N_2 and CO_2 saturated conditions.

**Compound**	**Under N_2**		**Under CO_2**	
	**E_p(1st)**	**E_p(2nd)**	**E_p(1st)**	**E_p(2nd)**
AQ	-0.73 V	-1.39 V	-0.72 V	-0.89 V
1-OH-AQ	-0.59 V	-1.13 V	-0.56 V	-0.85 V
1,4-OH-AQ	-0.45 V	-0.98 V	-0.47 V	-0.95 V
1-NH_2-AQ	-0.80 V	-1.40 V	-0.84 V	-0.94 V
1,4-NH_2-AQ	-0.96 V	-1.45 V	-0.92 V	-0.92 V
1-NH_2-4-OH-AQ	-0.72 V	-1.24 V	-0.73 V	-0.98 V

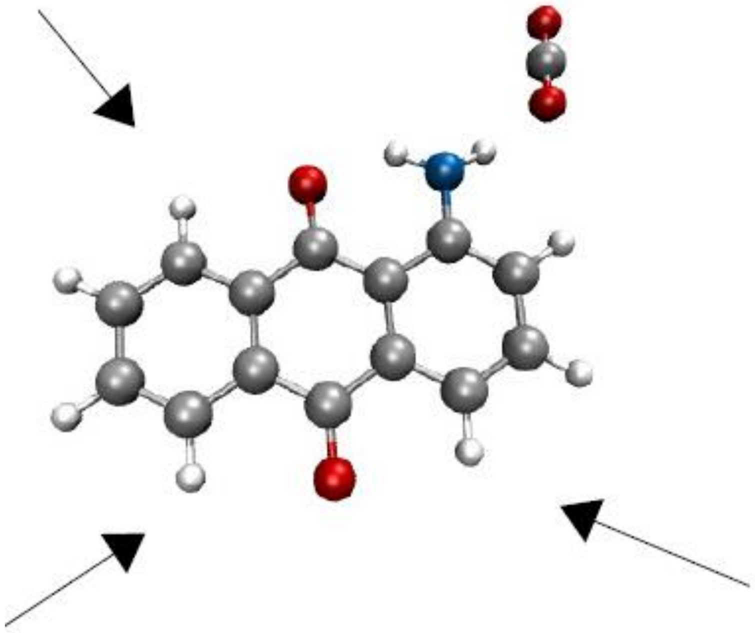

Figure S14: Illustration of the possible four approaching sites for coordinating CO_2 interactions exemplary shown with 1-NH_2-AQ.
